# Extracellular Vesicle Preparation and Analysis: A State‐of‐the‐Art Review

**DOI:** 10.1002/advs.202401069

**Published:** 2024-06-14

**Authors:** Zesheng Wang, Xiaoyu Zhou, Qinglong Kong, Huimin He, Jiayu Sun, Wenting Qiu, Liang Zhang, Mengsu Yang

**Affiliations:** ^1^ Department of Precision Diagnostic and Therapeutic Technology City University of Hong Kong Shenzhen Futian Research Institute Shenzhen Guangdong 518000 P. R. China; ^2^ Department of Biomedical Sciences and Tung Biomedical Sciences Centre City University of Hong Kong Hong Kong 999077 P. R. China; ^3^ Key Laboratory of Biochip Technology Biotech and Health Centre Shenzhen Research Institute of City University of Hong Kong Shenzhen 518057 P. R. China; ^4^ The Second Department of Thoracic Surgery Dalian Municipal Central Hospital Dalian 116033 P. R. China

**Keywords:** artificial intelligence, disease diagnosis, extracellular vesicles, single EV

## Abstract

In recent decades, research on Extracellular Vesicles (EVs) has gained prominence in the life sciences due to their critical roles in both health and disease states, offering promising applications in disease diagnosis, drug delivery, and therapy. However, their inherent heterogeneity and complex origins pose significant challenges to their preparation, analysis, and subsequent clinical application. This review is structured to provide an overview of the biogenesis, composition, and various sources of EVs, thereby laying the groundwork for a detailed discussion of contemporary techniques for their preparation and analysis. Particular focus is given to state‐of‐the‐art technologies that employ both microfluidic and non‐microfluidic platforms for EV processing. Furthermore, this discourse extends into innovative approaches that incorporate artificial intelligence and cutting‐edge electrochemical sensors, with a particular emphasis on single EV analysis. This review proposes current challenges and outlines prospective avenues for future research. The objective is to motivate researchers to innovate and expand methods for the preparation and analysis of EVs, fully unlocking their biomedical potential.

## Graphical abstract

This review provides an overview of the biogenesis, composition, and various sources of extracellular vesicles (EVs), and discusses current methods for their preparation and analysis. Particular focus is given to the latest technologies, including innovative microfluidic designs, new electrochemical sensors, nanotechnology, and artificial intelligence. Additionally, it explores recent advancements in single EV research to study the heterogeneity of EVs.

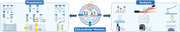

## 1 Introduction

In 1983, Harding et al. and Johnstone et al. independently discovered that reticulocytes secrete small vesicles to transport transferrin to the extracellular space during their maturation process.^[^ ^1^ ^]^ Though these small vesicles were initially dismissed as cellular waste and overlooked by the scientific community, the two seminal works laid a robust foundation for the subsequent advancement in EV research. The term ‘exosome’ was first introduced by Trams’ group in 1981.^[^ ^2^ ^]^ However, it was initially employed to describe vesicles shed from the cell membrane, a definition that differs from its current usage. Johnstone et al. later refined this definition, applying the term ‘exosome’ specifically to extracellular vesicles that are released following the fusion of multivesicular bodies (MVB) with the plasma membrane.^[^ ^3^ ^]^ This revised nomenclature has been sustained and received endorsement from the International Society for Extracellular Vesicles (ISEV) as a term specific to a subpopulation of EVs.^[^ ^4^ ^]^ As a rapidly evolving filed, the need for standardized terminology became urgent. Gyorgy et al. proposed that structures characterized by a non‐replicable lipid bilayer membrane should be termed ‘extracellular vesicles’.^[^ ^5^ ^]^ This terminology was subsequently adopted in the guidelines known as the Minimal Information for Studies of Extracellular Vesicles (MISEV).^[^ ^4^ ^]^

The realm of EV research has undergone significant paradigmatic shifts since 1996, evolving from perceiving EVs as mere cellular debris to recognizing them as pivotal entities with substantial functional implications. This transformation was catalyzed by Raposo et al.’s pioneering work, which demonstrated that B lymphocytes could secrete exosomes capable of antigen presentation to T cells, thereby initiating immunological responses.^[^ ^6^ ^]^ A following study by Zitvogel et al. in 1998 further revealed that dendritic cells also secreted exosomes with antigen‐presenting properties, contributing to anti‐tumor activities.^[^ ^7^ ^]^ Moreover, in 2007, Valadi et al. discovered that exosomes are rich in active RNA molecules that can be conveyed to recipient cells, signifying a pivotal shift in the field's academic perspective toward the recognition of exosomes as critical mediators of intercellular genetic material exchange.^[^ ^8^ ^]^ In light of these discoveries and the burgeoning scholarly output on EVs, the ISEV was founded in 2011 to promote global research collaboration and academic exchange. Further validation of the EVs field's significance came in 2013 when the Nobel Prize in Physiology or Medicine was awarded to James E. Rothman, Randy W. Schekman, and Thomas C. Südhof for elucidating the regulatory mechanisms of intracellular vesicles transport.^[^ ^9^ ^]^ This recognition led to rapid advancements in EV research and garnered interest from academic investigators and potential investors.

The advancing field of EVs has broadened our knowledge, revealing EVs as carriers of diverse bioactive molecules like proteins, nucleic acids (RNA and DNA), lipids and metabolites, critical for intercellular communication.^[^ ^10^ ^]^ Upon release into the extracellular environment, EVs exchange biological information and transfer through mechanisms such as autocrine and paracrine signaling, which influence both physiological and pathological cellular states.^[^ ^11^ ^]^ They are involved in the development and progression of myriad critical diseases such as cancer,^[^ ^12^ ^]^ cardiovascular disorders,^[^ ^13^ ^]^ diabetes,^[^ ^14^ ^]^ and neurological disease.^[^ ^15^ ^]^ Given that EVs are rich in various bioactive molecules and are widely present in various bodily fluids such as blood,^[^ ^16^ ^]^ urine,^[^ ^17^ ^]^ and saliva,^[^ ^18^ ^]^ these characteristics make EVs a promising biomarker for disease diagnosis.^[^ ^19^ ^]^ In 2016, the first EV‐based tumor diagnostic product, ExoDx™ Lung (ALK), received U.S. Food and Drug Administration (FDA) approval for clinical use, enabling the detection of exosomal mRNA mutations in patients with non‐small cell lung cancer.^[^ ^20^ ^]^ Following this, the ExoDx^TM^ Prostate (EPI), a urinary exosomes liquid biopsy product for prostate cancer patients, was also introduced to the market.^[^ ^21^ ^]^ Beyond their diagnostic utility, recent years have also witnessed an emergence of research employing various cell‐derived EVs for therapeutic purposes, revealing their unique advantages as drug carriers and therapeutic agents.^[^ ^22^ ^]^ For instance, EVs derived from mesenchymal stem cells hold significant research implications in neurodegenerative conditions and tissue regeneration.^[^ ^23^ ^]^ Unlike artificially synthesized carriers like liposomes, EVs offer superior biocompatibility and safety, allowing targeted modifications and drug loading to tailor therapeutic strategies for specific diseases. Currently, drug loading in EVs is mainly conducted through exogenous and endogenous methods, a detailed discussion of which can be found in other comprehensive reviews.^[^ ^24^ ^]^

Although EVs present immense potential in biomedical fields, their clinical application hinges on effective preparation and analysis. With the escalating interest in EV research, an ever‐increasing number of methodologies for preparation and analysis are being reported. Established preparation techniques include ultracentrifugation,^[^ ^25^ ^]^ density gradient centrifugation,^[^ ^26^ ^]^ size‐exclusion chromatography,^[^ ^27^ ^]^ ultrafiltration,^[^ ^28^ ^]^ polymer precipitation,^[^ ^29^ ^]^ and immunomagnetic beads.^[^ ^30^ ^]^ Additionally, emerging techniques have further expanded the repertoire, encompassing microfluidics,^[^ ^31^ ^]^ tangential flow filtration,^[^ ^32^ ^]^ asymmetric flow field‐flow fractionation,^[^ ^33^ ^]^ and anion exchange chromatography.^[^ ^34^ ^]^ Following preparation, EVs are characterized to ascertain their biophysical and biochemical properties. Biophysical attributes, such as size, shape, and mechanical properties, are evaluated through methods like nanoparticle tracking analysis,^[^ ^35^ ^]^ dynamic light scattering,^[^ ^36^ ^]^ transmission electron microscopy,^[^ ^37^ ^]^ scanning electron microscopy,^[^ ^38^ ^]^ cryogenic electron microscopy,^[^ ^39^ ^]^ atomic force microscopy,^[^ ^40^ ^]^ and tunable resistive pulse sensing.^[^ ^41^ ^]^ Biochemical analysis, focusing on proteins, nucleic acids, lipids and metabolites, is conducted using methods like western blotting,^[^ ^42^ ^]^ enzyme‐linked immunosorbent assays,^[^ ^43^ ^]^ mass spectrometry,^[^ ^44^ ^]^ real‐time polymerase chain reaction,^[^ ^45^ ^]^ surface plasmon resonance,^[^ ^46^ ^]^ and surface‐enhanced Raman scattering.^[^ ^47^ ^]^ Upon reviewing diverse methodologies for the preparation and analysis of EVs, it becomes clear that ensuring the safety and quality of EV products is of paramount importance. Each method presents distinct benefits in processing EVs but also brings about variables that may profoundly affect the quality and safety of the end products, especially in the context of therapeutic uses.^[^ ^48^ ^]^ Therefore, a stringent focus on safety and quality management is essential, which includes careful selection of source cells, refinement of culture conditions, uniformity in the preparation steps, and accuracy in analytical procedures.^[^ ^49^ ^]^

Given the rapid emergence of new methods for EV preparation and analysis, timely reviews of these state‐of‐the‐art techniques can offer a comprehensive reference to researchers either already engaged in or considering entry into EV research. Although existing review articles have provided insights into specific aspects of EV research, they either primarily focus on microfluidic platforms^[^ ^50^ ^]^ or solely offer reviews on separation^[^ ^51^ ^]^ or characterization.^[^ ^52^ ^]^ However, a more encompassing review is still relatively scarce. To address these gaps, this presented review commences with the fundamental concepts related to EV biogenesis, composition, and sources, followed by a comprehensive survey of preparation and characterization methods. Particular emphasis is placed on elucidating cutting‐edge technologies, such as those based on both microfluidic and non‐microfluidic platform, including but not limited to DNA nanotechnology and emerging peptide methods. Furthermore, this review highlights recent progress in the development of novel electrochemical sensors and the application of artificial intelligence for EV analysis, alongside advanced analytical strategies for investigating single EV. Finally, this review not only identifies existing challenges in the field but also outlines potential avenues for future research. The intended impact of this review is to stimulate the development of effective, clinically applicable methods to fully exploit extensive biomedical potential of EVs.

## 2 EV Fundamentals

### 2.1 EV Biogenesis

According to the MISEV2023 guideline on EV nomenclature, “extracellular vesicles” (EVs) are identified as particles secreted by cells, enclosed by a lipid bilayer, without the capacity for self‐replication.^[^ ^53^ ^]^ An increasing number of research suggests that EVs display considerable heterogeneity, covering both in size and distinct biological characteristics and complex formation mechanisms.^[^ ^54^ ^]^ Based on differences in their biogenesis mechanisms and biological characteristics, the current classification system primarily distinguishes EVs into three key types: exosomes, microvesicles, and apoptotic bodies.^[^ ^55^ ^]^ Exosomes, ranging in diameter from 40 to 200 nm (or 30 to 150 nm),^[^ ^56^ ^]^ are formed by fusing multivesicular bodies with the cell membrane. Microvesicles, which have diameters between 200 and 1000 nm (or 100 to 1000 nm),^[^ ^56^ ^]^ are generated through the direct budding of the cell membrane. Apoptotic bodies, encapsulating cytoplasm and organelles within a membrane, are created during cellular apoptosis and measure between 500 and 2000 nm in diameter (or 50 to 5000 nm).^[^ ^56^, ^57^ ^]^ The formation mechanisms of EVs are intricate and not fully understood. Here is a concise overview of the widely accepted and extensively researched mechanisms for the three types of EVs.

**Figure 1:**
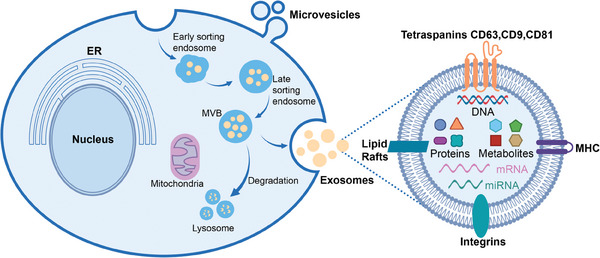
An overview of the biogenesis pathways for two primary types of EVs (exosomes and microvesicles), along with a structural schematic of exosomes. ER: endoplasmic reticulum; MVB: multivesicular bodies; MHC: major histocompatibility complex I/II.

To begin with exosomes, whose formation contains a series of precise cellular regulatory processes (**Figure** **1**).^[^ ^58^ ^]^ During the initial stages, invagination of the cell membrane results in the formation of endocytic vesicles. Subsequently, these vesicles undergo fusion processes to create early endosomes.^[^ ^59^ ^]^ Following their formation, the early endosomes evolve by encapsulating intracellular constituents, transitioning into multivesicular vesicles recognized as late endosomes. As these vesicles integrate intracellular proteins, enzymes, and various biomolecules, they culminate in their maturation to MVB.^[^ ^60^ ^]^ Lastly, mature MVB can either fuse with the cell membrane to secrete exosomes into the extracellular space or fuse with lysosomes for degradation.^[^ ^61^ ^]^ The specificity of this pathway is underscored by the critical role of the endosomal sorting complex required for transport (ESCRT) in exosomes formation.^[^ ^62^ ^]^ Interestingly, recent insights have unveiled an ESCRT‐independent mechanism, further illustrating the intricate nature of exosomes biogenesis.^[^ ^63^ ^]^ In contrast, microvesicles are formed directly from the plasma membrane, through a process characterized by the outward budding.^[^ ^64^ ^]^ Mechanistic studies reveal that the biogenesis of microvesicles involves a series of intracellular molecular rearrangements, including alterations in lipid composition, protein constituents, and calcium ion concentrations.^[^ ^61^, ^65^ ^]^ The third type of EVs is apoptotic bodies, generally characterized as vesicles with a diameter not exceeding 5 µm. These are formed during cellular apoptosis when the cell membrane undergoes invagination. Then, cellular components such as cytoplasm and organelles are encapsulated, leading to the formation of these vesicular structures through a process of membrane blebbing and detachment.^[^ ^66^ ^]^ The formation of apoptotic bodies is a strictly controlled process, yet the key mechanisms behind it remain unclear and are not discussed in detail here.^[^ ^67^ ^]^

### 2.2 EV Compositions

EVs contain diverse bioactive molecules mainly consisting of proteins, nucleic acids, lipids and metabolites (Figure 1).^[^ ^68^ ^]^ Recent technological advances in mass spectrometry and high‐throughput sequencing enabled comprehensive analysis of EV contents, thereby illuminating their roles and regulatory mechanisms in physiological and pathological states. Databases like Vesiclepedia,^[^ ^69^ ^]^ ExoCarta^[^ ^70^ ^]^ and EVpedia^[^ ^71^ ^]^ offer updated inventories of EV components, serving as valuable references for researchers and clinicians.

EVs harbor proteins that predominantly fall into two main categories.^[^ ^72^ ^]^ The first category consists of structural and ubiquitously present proteins widely distributed on the surface or within the lumen of EVs. This group encompasses cytoskeletal components such as microtubule proteins, actin, and filament‐binding proteins, as well as members of the tetraspanin superfamily like CD9, CD63, CD81, and CD82.^[^ ^73^ ^]^ Further, it includes proteins such as Annexins, Flotillin, Alix, and TSG101, pivotal for membrane fusion and cellular trafficking,^[^ ^58^, ^74^ ^]^ while heat shock proteins HSP70 and HSP90 play roles in stress responses.^[^ ^75^ ^]^ The second category is linked to the cellular origin of the EVs. For instance, EVs derived from antigen‐presenting cells, such as macrophages and dendritic cells, are enriched with molecules like MHC‐I and MHC‐II.^[^ ^76^ ^]^ Additionally, EVs originating from tumor cells may display overexpressed markers like TGF‐β and EpCAM.^[^ ^77^ ^]^ Investigating the proteins of EVs serves a dual purpose: it not only enhances our understanding of their roles in disease onset and progression but also aids in the identification of potential biomarkers for early diagnosis and prognostic evaluation of diseases.^[^ ^78^ ^]^ Beyond proteins, EVs carry a spectrum of nucleic acids, including genomic DNA, mitochondrial DNA, and a plethora of RNA species like mRNA, miRNA, lncRNA, and cirRNA.^[^ ^79^ ^]^ These nucleotide fragments, often ≈200 base pairs in size, have the capability to translate into functional proteins, thereby influencing the recipient cells' biological functions.^[^ ^80^ ^]^ Of particular interest in current research is EV‐derived RNA. With the advent of advanced RNA sequencing techniques, the biological functions of various RNAs within EVs have been uncovered, revealing their regulatory roles in multiple biological processes.^[^ ^81^ ^]^ Moreover, the RNA content within EVs is being explored as a promising new biomarker for disease diagnosis.^[^ ^82^ ^]^ In addition to their nucleic acid content, EVs are characterized by specialized membrane structures rich in unique lipids such as cholesterol, phospholipids, phosphatidylethanolamine, diglycerides, and phosphatidylserine. Notably, the lipidomic profile of these vesicles is subject to variation, contingent upon the cell type from which they originate.^[^ ^83^ ^]^ These lipids not only participate in the biogenesis and uptake of EVs but also act as vital bioactive molecules involved in immune surveillance, tumor microenvironment modification, inflammation regulation, and various biological processes.^[^ ^84^ ^]^ Analogous to the proteins and RNA in EVs, the lipid constituents within these vesicles have the potential to act as biomarkers reflecting abnormalities in lipid metabolism within the organism.^[^ ^85^ ^]^ Moving beyond the lipid components, the metabolites within EVs provide a window into the metabolic state of their cells of origin. These metabolites play a crucial role in influencing biological functions and the progression of diseases, emphasizing their significance in the diagnostic and therapeutic applications of EVs.^[^ ^86^ ^]^ Analyzing these metabolites allows us to enhance our understanding of EV composition and propel forward their potential in clinical settings.^[^ ^87^ ^]^

### 2.3 EV Sources

EVs are ubiquitously secreted by numerous cell types and can be obtained from different biological sources, such as human biofluids, tissues, and cell culture supernatant.^[^ ^88^ ^]^ For a comprehensive understanding of various sources, the MISEV2023 guideline offers a detailed overview.^[^ ^53^ ^]^ The following discussion will concentrate on the sources that have garnered the most extensive study. First, blood‐derived EVs are a focal point of current research, particularly in liquid biopsy, a significant method for tumor detection using blood samples.^[^ ^89^ ^]^ However, the biochemical complexity of blood, which is enriched with cellular components, free nucleic acids, and lipoproteins, introduces substantial obstacles in the effective preparation of EVs. Specifically, these challenges arise from the overlapping size and density between lipoprotein particles and EVs.^[^ ^11a^ ^]^ In parallel, there is a burgeoning interest in EVs purified from human urine, primarily due to the non‐invasive nature of urine as a liquid biopsy source. Such urinary EVs are predominantly sourced from renal and bladder tissues.^[^ ^17^, ^90^ ^]^ However, much like blood, urine also presents a complex biochemical composition, replete with metabolized inorganic and organic molecules, urinary‐specific proteins, and bacterial components, adding further intricacy to their purification.^[^ ^91^ ^]^ EVs can also be isolated from other human fluids or tissues, such as saliva, tears, feces, and cerebrospinal fluid.^[^ ^92^ ^]^ Another extensively investigated source of EVs is derived from cell culture supernatant and these cells are frequently of mammalian origin, such as human or mouse. Various cells like mesenchymal stem cells, cancer cells, dendritic cells, immune cells, etc., are cultured without serum or with EV‐depleted serum using 2D or 3D cultivation methods.^[^ ^93^ ^]^ During culture, these cells not only secrete EVs but also other metabolites such as soluble proteins and cytokines, illustrating the diverse nature of EV production and the complexity involved in studying them.^[^ ^88^ ^]^

Beyond the well‐researched domains of human biological fluids and cell culture supernatant as sources of EVs, increasing scholarly focus is being directed toward EVs derived from cow's milk, plants, and bacteria. These novel sources present economical and sustainable advantages, offering the potential for large‐scale production at lower cost and higher yield of EVs.^[^ ^94^ ^]^ Cow's milk‐derived EVs stand out for their cost‐effectiveness and ease of accessibility, eliminating the need for cell culture and thus representing a promising alternative for large‐scale production.^[^ ^95^ ^]^ Carobolante et al. have illustrated the potential of milk‐derived EVs in enhancing the oral bioavailability of drugs, thus acting as efficient carriers for bioactive compounds.^[^ ^96^ ^]^ Additionally, Munagala et al. have explored the customization of these EVs with specific ligands, such as folic acid, for targeted approaches in cancer therapy.^[^ ^97^ ^]^ Building upon the inherent medicinal attributes of many plants‐such as anti‐inflammatory, anti‐tumor, and anti‐aging properties‐there has been growing interest in plant‐derived EVs. To date, EVs have been successfully isolated from ginger, grapes, grapefruit, lemon, broccoli, apples, ginseng, coconut, blueberries.^[^ ^98^ ^]^ Notably, Sahin et al. demonstrated that wheatgrass‐sourced EVs could promote skin regeneration by inducing the proliferation of various cell types in a dose‐dependent manner.^[^ ^99^ ^]^ Moreover, plant‐derived EVs can be engineered to carry proteins, nucleic acids, and other bioactive molecules for therapeutic applications.^[^ ^100^ ^]^ In the bacterial realm, EVs are primarily investigated in the context of Gram‐negative bacteria, specifically their outer membrane vesicles (OMVs). These OMVs are rich in a variety of biological components, including proteins, lipids, and nucleic acids, and play specialized roles in both intra‐bacterial and bacteria‐host interactions.^[^ ^101^ ^]^

While this review will not delve into the specific preparation and analysis techniques for EVs from each unique source, it is essential to highlight that methodological similarities exist across these origins, allowing for cross‐referencing of methods. The principal focus of this review will be on EVs derived from human biological fluids and cell culture supernatant, given their extensive research coverage and closer relevance to clinical applications.

## 3 EV Preparation Techniques

**Figure 2:**
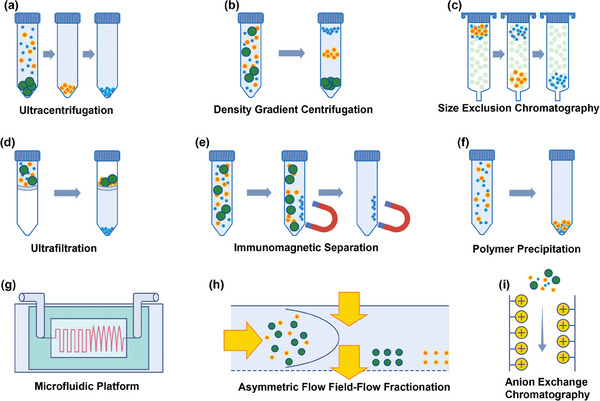
Illustration of commonly used techniques for EV preparation. These techniques include a) ultracentrifugation, which leverages high‐speed centrifugal forces; b) density gradient centrifugation, which separates EVs based on their buoyancy; c) size‐exclusion chromatography, for isolating EVs by size; d) ultrafiltration, a method that uses membrane filters for size‐based separation; e) immunomagnetic separation, which targets specific EV markers with magnetic beads; f) polymer precipitation, where polymers are used to precipitate EVs out of solution; g) microfluidics platforms, offering precise manipulation of fluids to isolate EVs; h) asymmetric flow field‐flow fractionation, which separates particles based on their hydrodynamic size in a flow; i) anion exchange chromatography, which isolates EVs based on charge differences.

EV preparation is a crucial prerequisite for both basic scientific research and translational clinical applications. This process presents a complex challenge, for the physicochemical characteristics of EVs often overlap with those of other biological entities like lipoproteins and protein complexes.^[^ ^11^, ^102^ ^]^ Different preparation methods can variably impact the concentration and purity of EVs, thereby affecting downstream analyses.^[^ ^103^ ^]^ Consequently, selecting a suitable preparation technique is vital for ensuring research quality. Currently, a variety of methods have been developed (as depicted in **Figure** **2**), each with its own set of advantages and limitations. However, a universally accepted standard for EV preparation is elusive, and achieving 100% purity is technically impossible.^[^ ^88^ ^]^ Therefore, the choice of preparation methodology should be tailored to align with the specific aims and requirements of the research at hand.

### 3.1 Conventional Methods

Ultracentrifugation (UC), also known as differential centrifugation, is the most frequently reported methodology in published research on EVs.^[^ ^104^ ^]^ This technique is particularly well‐suited for isolating EVs from larger volumes, such as cell culture supernatant. The foundational premise lies in the significant differences in sedimentation rates between particles of varying diameters. Larger particles precipitate more rapidly, while smaller ones require greater centrifugal forces or extended centrifugation periods for sedimentation. The yield and purity of EVs can be significantly impacted by various parameters during the centrifugation process, specifically the rotor type and centrifugation duration.^[^ ^105^ ^]^ Théry et al. have outlined a comprehensive ultracentrifugation protocol for EV preparation that involves the sequential elimination of intact cells, dead cells, cell debris, and larger particulate impurities.^[^ ^25^ ^]^ EV preparation steps: 1) Centrifuge supernatant at 300 g, 10 min to clear dead cells. 2) Centrifuge at 2000 g, 15 min to remove debris. 3) Centrifuge at 10 000 g, 30 min to discard large vesicles. 4) Filter at 0.22 µm, then ultracentrifuge at 100 000 g, 120 min. 5) Resuspend in PBS, ultracentrifuge again, and prepare the pellet for immediate use or store at −80 °C for future use. UC is chosen for its widespread acceptance in EV isolation, offering a balance between efficiency and purity, and its versatility in capturing a broad spectrum of EV sizes for various applications.^[^ ^106^ ^]^ However, it's important to note that while UC can yield relatively high concentration of EVs, it may also co‐isolate protein aggregates or other vesicles, which could affect the purity of the EV sample. This characteristic of UC necessitates subsequent purification steps or the combination with other techniques, such as size‐exclusion chromatography (SEC), to enhance purity.^[^ ^107^ ^]^

Density gradient centrifugation (DG) combines ultracentrifugation with a gradient medium, typically sucrose or iodixanol, to distinguish EVs from non‐vesicular particles.^[^ ^108^ ^]^ Traditionally, the method requires an initial ultracentrifugation step to pre‐concentrate EVs. Following this, the concentrated sample is resuspended in PBS and then subjected to additional purification through sucrose or iodixanol density gradient ultracentrifugation. DG is selected for its ability to separate EVs based on density, allowing for the isolation of distinct EV populations with minimal contamination.^[^ ^106^ ^]^ This method is particularly useful for studies requiring high purity EVs for functional assays. However, the process can be time‐consuming and may lead to lower yields due to the multiple centrifugation steps required.^[^ ^109^ ^]^ In a comprehensive study, Li et al. demonstrated the efficacy of iodixanol‐based density centrifugation, marking a significant improvement over traditional ultracentrifugation methods in terms of both EV recovery and purity.^[^ ^110^ ^]^ Simultaneously, Mohanty et al. have optimized a one‐step sucrose cushion ultracentrifugation protocol to eliminate the need for pre‐concentration.^[^ ^111^ ^]^ In their streamlined approach, the biological sample is directly layered over a sucrose cushion, followed by centrifugation at 100 000 g and 4 °C for 90 min. After discarding the supernatant and collecting the sucrose cushion, a purified fraction is diluted in PBS for a second centrifugation under identical conditions. Complementing these advances, Hendrix et al. applied density gradient centrifugation to purify urinary EVs for proteomic analysis. Their findings confirmed the efficacy of this approach in separating EVs from protein contaminants, highlighting its potential for specific applications that require high‐purity EV samples.^[^ ^112^ ^]^

Size‐Exclusion Chromatography (SEC) offers a robust technique for molecules separation based on size or molecular weight. The mechanism relies on the porous polymer microspheres packed into the chromatographic column. Through this column, smaller molecules migrate more slowly than their larger counterparts.^[^ ^113^ ^]^ The attractiveness of SEC lies in its capacity to efficiently eliminate protein contaminants and isolate EVs by size, while maintaining their biological integrity. This results in relatively high purity samples, suitable for subsequent biological analyses and ideal for functional studies and omics research. However, the primary drawback of the technique is the potential dilution of EV samples, which may necessitate post‐isolation concentration steps.^[^ ^109^, ^114^ ^]^ Considering these fundamental principles, María et al. meticulously optimized SEC parameters, focusing on the pore size and the degree of crosslinking in agarose fillers. Their research pinpointed optimal conditions for effectively separating EVs from lipoprotein contaminants.^[^ ^115^ ^]^ Furthermore, Mariele et al. refined the approach by combining SEC with ultrafiltration (UF) to efficiently isolate intact EVs from blood samples, achieving notable results.^[^ ^116^ ^]^ This SEC‐UF synergy was further validated by Davies et al., who confirmed its effectiveness in preparing EVs sourced from mouse skeletal muscle myoblasts and noted enhancements in both sample throughput and purity.^[^ ^117^ ^]^ Further research indicates that SEC method also plays a facilitative role in the large‐scale production of EVs for therapeutic applications. Jung et al. reported a streamlined protocol for large‐scale EV production. It starts with filtration to remove large particles, followed by polyethylene glycol (PEG) precipitation to concentrate the sample. The final purification is achieved through SEC, efficiently isolating EVs for therapeutic purposes.^[^ ^118^ ^]^ Additionally, it's worth mentioning that commercial solutions have also been developed to leverage SEC's benefits for EVs isolation. Prominent kits on the market include qEV by iZON Science, EVSecond from GL Sciences, ExoLutE by Rosetta Exosome Company in Korea, and HansaBioMed's PURE‐EVs.^[^ ^119^ ^]^

Polymer Precipitation, originally developed for purifying viral particles,^[^ ^120^ ^]^ has also been adapted for EV preparation due to their shared physicochemical properties. The technique predominantly uses PEG for precipitation. PEG, a hydrophilic compound, attracts water molecules, leading to the reduced solubility and aggregation of EVs, facilitating their precipitation upon centrifugation.^[^ ^121^ ^]^ The efficiency of PEG precipitation depends on various factors, including the PEG's concentration and molecular weight, which typically range from 6,000 to 20,000, as well as external conditions like ionic strength, solution pH, and temperature.^[^ ^122^ ^]^ This method is efficient for processing large volumes and yields a high quantity of EVs. However, the major drawback is the co‐precipitation of non‐EV proteins and other particulates, resulting in lower purity. This necessitates additional purification steps for applications requiring high purity EVs, such as molecular analysis and therapeutic uses.^[^ ^113^ ^]^ The versatility of the PEG precipitation method is demonstrated by its successful integration with SEC. Rajesh et al. successfully employed this hybrid technique for preparing EVs from pericardial fluid, especially for samples with low EV concentrations.^[^ ^123^ ^]^ Similarly, Eduardo et al. merged PEG precipitation with SEC to isolate EVs for quantitative proteomic analysis of distinct EV subpopulations.^[^ ^124^ ^]^ Expanding upon these findings, Shih et al. conducted a targeted optimization analysis focusing on the PEG concentration necessary for precipitation. Their work established that an 8% PEG solution is efficacious in the purification of EVs originating from oral squamous cell carcinoma.^[^ ^29^ ^]^ Capitalizing on PEG's versatility and enrichment capabilities, a host of commercial EV preparation kits have emerged. Notably, these are Exo‐spin Isolation Kit by Cell Guidance Systems, ExoQuick Exosome Precipitation from System Biosciences, miRCURY Exosome Isolation Kit by Exiqon, and Invitrogen's Total Exosome Isolation Reagent.^[^ ^119^ ^]^

Ultrafiltration (UF) method is based on molecular size separation, sharing fundamental principles with traditional membrane filtration techniques.^[^ ^125^ ^]^ It uses external driving forces to separate molecules based on pore size. This method effectively concentrates EVs from large volumes quickly, leveraging the principle that larger molecules are retained while smaller ones are allowed to pass through. Despite its efficiency, UF's limitation lies in the potential loss of smaller EVs and the risk of contamination with similarly sized proteins, making it best suited for applications that require moderate purity and prioritize high throughput.^[^ ^119^ ^]^ In UF, two primary configurations are employed: dead‐end filtration and tangential flow filtration (TFF).^[^ ^113^, ^126^ ^]^ TFF, in particular, has gained prominence due to its design, which aligns fluid flow perpendicular to the filtration direction, with a tangential flow that reduces membrane fouling. This configuration is especially compatible with the particle size distribution of EVs, offering a rapid and efficient separation method.^[^ ^127^ ^]^ Notable research has demonstrated the potential of TFF in EV isolation. Wolfram et al. emphasized TFF's ability to isolate high‐quality cell‐derived vesicles from large volumes, presenting it as a scalable and gentler alternative to ultracentrifugation.^[^ ^32^ ^]^ In addition, the integration of TFF with microfluidic technology, as explored by Liu et al., resulted in a dual‐TFF microfluidic device that significantly improved EV purity and yield from cell supernatant and human serum.^[^ ^128^ ^]^ Young et al. further enhanced the tangential flow ultrafiltration method by integrating electrophoretic oscillation to minimize fouling and pore‐clogging from larger biological particles. Finally, the method achieved high purity and integrity of bovine milk‐derived EVs.^[^ ^129^ ^]^ Currently, commercial kits such as ExoMir (Bioo Scientific, Austin, TX, USA) utilize membrane filtration techniques for EV preparation.^[^ ^130^ ^]^

Anion Exchange Chromatography (AEC) serves as a robust method for the preparation of EVs, leveraging the inherent negative charge of EVs to interact with charged resins within the chromatographic column.^[^ ^131^ ^]^ This technique involves several critical steps to ensure the purity and integrity of the isolated EVs. Initially, EV‐containing samples are prepared by removing unrelated elements, such as cellular debris, through low‐speed centrifugation. Then, the cleaned sample is loaded into a column filled with cation‐exchange resins, where the negatively charged EVs bind selectively to the resins. Following this binding stage, elution is carried out by altering the column's salt concentration and pH levels, thereby separating the bound EVs from other charged particles and contaminants.^[^ ^34^, ^131^, ^132^ ^]^ This method can achieve high‐purity EV isolation by exploiting the charge properties of EVs. It allows for the separation of EVs from proteins and other charged particles. However, the process can be more complex and time‐consuming, requiring optimization to avoid EV loss due to undesired interactions with the column matrix.^[^ ^119^, ^133^ ^]^ Multiple studies have validated the effectiveness of AEC. For example, Steven et al. successfully combined TFF with AEC to purify bacterial EVs.^[^ ^133^ ^]^ Similarly, Naohiro et al.’s work with AEC showcased the method's versatility in isolating cytotoxic T‐lymphocyte EVs, revealing how manipulating NaCl concentrations during elution can influence the nature of the isolated EVs.^[^ ^34^ ^]^ In a distinct approach, Ana et al. applied nucleases before AEC purification for human mesenchymal stem cell‐derived EVs, achieving a remarkable 98% reduction in protein and DNA contaminants.^[^ ^134^ ^]^ Complementing AEC, cation‐exchange chromatography offers another avenue for removing positively charged impurities from EV samples.^[^ ^134^ ^]^

Asymmetrical Flow Field‐Flow Fractionation (AF4) is a field‐flow fractionation technique initially proposed by Wahlund and Giddings in 1987.^[^ ^135^ ^]^ In AF4, the bottom of the separation channel is lined with a semi‐permeable membrane with a set size‐exclusion limit. This design allows smaller molecules and solvent to pass through while retaining larger particles.^[^ ^136^ ^]^ A vital aspect of the technique is the applied crossflow, which is perpendicular to the sample flow's direction. This crossflow, in balance with the sample's natural diffusion forces, enables the segregation of particles primarily on the basis of their diffusion coefficients. Consequently, particles with higher diffusion rates, typically smaller in size, elute from the system earlier than larger, slower‐diffusing counterparts. This mechanism underscores AF4's ability to achieve high‐resolution separation, making it an attractive choice for isolating EVs with minimal stress, thereby preserving their structural and functional integrity due to the absence of a stationary phase.^[^ ^137^ ^]^ AF4's efficacy extends to its capacity for examining EV heterogeneity and size distribution, providing a window into the complex nature of these particles. However, despite its advantages, the complexity of the AF4 method and the requirement for specialized equipment have limited its widespread application.^[^ ^33^, ^135^, ^137^ ^]^ The utility of AF4 for EV preparation has been underscored by multiple studies.^[^ ^138^ ^]^ Zhang et al. successfully employed the technique to segregate two distinct EV subpopulations based on their sizes.^[^ ^136^ ^]^ Another study by Zhong et al. combined AF4 with capillary electrophoresis to achieve size‐based, gentle, and rapid EV separation while effectively isolating them from proteins and high‐density lipoproteins.^[^ ^139^ ^]^ Rimsa et al. took this a step forward by tailoring an AF4 system specifically for EVs purification, optimizing particle confinement near the membrane to enable continuous flow operations.^[^ ^140^ ^]^ Such an innovation is particularly beneficial for processing high‐throughput, large‐volume samples, illustrating AF4's potential for scalability and efficiency in EV isolation.

**Figure 3:**
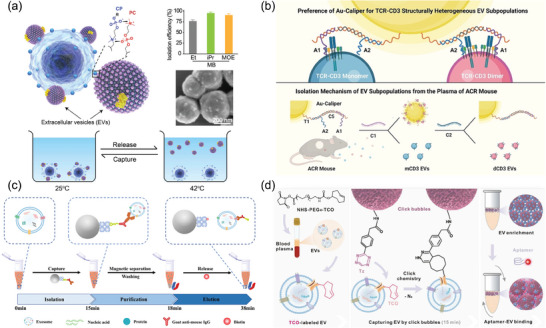
Latest reported affinity‐based methods for EV preparation include: a) The mechanism for adhesion of EVs to a magnetic bead coated with choline phosphate, utilizing the high‐affinity interaction between phosphatidylcholine and choline phosphate, followed by subsequent release after capture. Reproduced with permission.^[^ ^150^ ^]^ Copyright 2023, American Association for the Advancement of Science. b) Isolation mechanism for structurally heterogeneous TCR‐CD3 EV subpopulations. Reproduced with permission.^[^ ^152^ ^]^ Copyright 2023, Wiley‐VCH, GmbH. c) The exosome isolation process using the SIMI system. Reproduced with permission.^[^ ^153^ ^]^ Copyright 2023, American Chemical Society. d) EVs captured via a bioorthogonal click chemistry reaction. Reproduced with permission.^[^ ^155^ ^]^ Copyright 2023, Wiley‐VCH, GmbH.

Affinity‐based methods for preparing EVs hinge on specific biochemical interactions such as immunological antigen‐antibody pairings or ligand‐receptor affinities. These methods commonly use antibodies,^[^ ^141^ ^]^ aptamers,^[^ ^142^ ^]^ lipid moieties,^[^ ^143^ ^]^ peptides,^[^ ^144^ ^]^ and glycan‐based components^[^ ^145^ ^]^ as the basis for separation. The primary advantage of these methods is their ability to achieve highly specific isolation based on surface markers, ensuring very high purity of the isolated EVs. However, this specificity may come at the cost of lower yield and the potential alteration of the EVs' surface properties or functionality due to the binding process. Given these considerations, affinity purification is particularly well‐suited for research focused on specific EV populations but may face limitations in large‐scale applications because of cost and scalability challenges.^[^ ^146^ ^]^ Among the most prevalent are antibody‐based approaches, where antibodies are often conjugated with magnetic beads or immobilized on microfluidic chips.^[^ ^31^, ^141^ ^]^ The antibodies selected for these methods typically target transmembrane proteins like CD63, CD9, and CD81, as well as oncogenic markers such as CA‐125 and EpCAM.^[^ ^147^ ^]^ Aptamer‐based methods represent another growing field in affinity‐based EV isolation. The distinguishing feature of aptamers lies in their unique 3D structures, enabling strong and specific binding to designated target molecules. Their ease of modification and cost‐effectiveness make them an increasingly popular choice for EV isolation.^[^ ^142^, ^148^ ^]^ Additionally, Lipid‐based strategies utilize the lipid composition of EV membranes, which are rich in amphiphilic phospholipids, to facilitate isolation through hydrophobic interactions.^[^ ^143a^ ^]^ Recently, peptide‐based methods, involving biological macromolecules synthesized through amino acid condensation, have also emerged as promising techniques for EV isolation.^[^ ^144^ ^]^ Beyond proteins and lipids, glycan‐based components offer yet another strategy for EV preparation. The EV surface features a complex array of glycoconjugates, including O‐linked and N‐linked glycans, gangliosides, and other sugar moieties, thus expanding the scope for specific capture.^[^ ^145^ ^]^ Recent advancements in affinity‐based methods for EV isolation have shown remarkable progress. Liu et al. devised a dual‐switch peptide system sensitive to low pH levels, called D‐S pHLIP, specifically designed for targeting EVs from the tumor microenvironment (TME).^[^ ^149^ ^]^ This system incorporates two functional switches: one for installing D‐S pHLIP onto the EV membranes in the acidic TME and another hook‐shaped one to secure the peptide during systemic circulation. Similarly, the same group introduced magnetic beads functionalized with choline phosphate to isolate EVs (**Figure** **3**a).^[^ ^150^ ^]^ This approach takes advantage of the affinity between choline phosphate and EV membrane components for selective capture, allowing for easy release by raising the solution temperature. In their research, it was found that the separation efficiency is optimal when the alkyl substituent R on the choline phosphate monomer is an isopropyl group. Additionally, Sun et al. utilized a combination of L‐cysteine and titanium dioxide on magnetic nanoparticles for EV isolation, identifying a wealth of glycans and associated glycoproteins.^[^ ^151^ ^]^ Dai and collaborators introduced nucleic acid aptamer probes, designed to identify and isolate structurally diverse TCR‐CD3 exosomes subpopulations (Figure 3b).^[^ ^152^ ^]^ These probes, attached to gold nanoparticles, allowed for precise structural differentiation and revealed significant heterogeneity among the isolated EV subpopulations, underscoring the potential of monomeric CD3 EVs as biomarkers for acute cellular rejection. Peng et al. reported a Strep‐tag II system as a “cleavable bridge” to construct superparamagnetic nanoparticles modified with capturing antibody CD63 for EV separation (Figure 3c).^[^ ^153^ ^]^ Following capture, exosomes release was achieved through competitive interactions using the higher affinity between biotin and Strep‐Tactin, resulting in separation and release efficiencies of 82.5% and 62%, respectively. The field has also seen advancements in click chemistry for EV isolation. Zhu et al. utilized lipid‐based labeling combined with click chemistry, employing trans‐cyclooctene‐labeled lipids and tetrazine‐modified beads for efficient EV capture.^[^ ^154^ ^]^ Similarly, Yang et al. introduced a click bubble‐driven nucleic acid aptasensor, leveraging the cyclization reaction between trans‐cyclooctene and tetrazine for rapid EV enrichment (Figure 3d).^[^ ^155^ ^]^ Moreover, Tao and colleagues developed an amphiphilic dendritic supramolecular probe that integrates into EV lipid bilayers, enabling isolation through multivalent interactions.^[^ ^156^ ^]^ These examples illustrate the cutting‐edge developments in affinity‐based methods for EV isolation, showcasing their efficiency and specificity in EV research.

### 3.2 Advanced Methods

After discussing conventional methods for EV preparation, the focus shifts to advanced techniques, including novel microfluidic designs and nanotechnology. Microfluidics, often heralded as ‘lab‐on‐a‐chip’ technology, stands out for its ability to manipulate fluids with unparalleled precision at the microscale.^[^ ^157^ ^]^ This engineering discipline allows for the meticulous customization of channel attributes, including their length, diameter, composition, and surface properties‐such as the integration of specific antibodies for targeted interaction.^[^ ^158^ ^]^ This enables precise control over particulate behavior in the fluid medium.^[^ ^157b^ ^]^ Additionally, the technology can be augmented by external force fields like acoustic, magnetic, and electric fields to improve component separation and purification.^[^ ^159^ ^]^ With the advent of novel microfabrication techniques, the unique advantages of microfluidic platforms have gained prominence in biomedical applications. Specifically for EV separation, microfluidics offers key benefits over traditional approaches, such as reduced sample volumes, faster processing, and higher recovery rates. Concurrently, the swift advancement of nanotechnology has made significant strides in biomedicine and molecular diagnostics.^[^ ^160^ ^]^ The utilization of nanostructures and nanomaterials in EV isolation stands out for its effectiveness.^[^ ^161^ ^]^ Their superior performance in EV separation owes much to the substantial surface area‐to‐volume ratio of nanomaterials. This trait provides a plethora of binding sites, significantly increasing the potential for interaction with EVs and thus, enhancing the efficiency of their capture.^[^ ^162^ ^]^

#### 3.2.1 Novel Microfluidic Design Technologies

**Figure 4:**
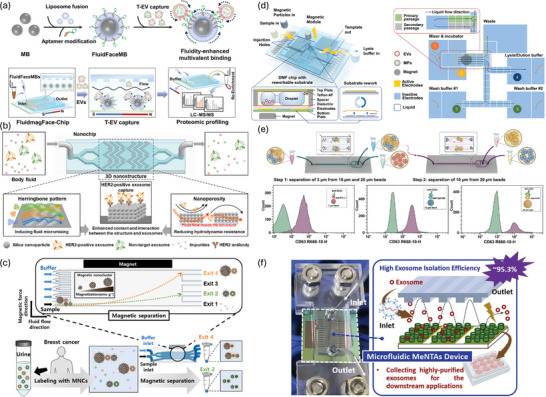
Cutting‐edge microfluidic techniques for EV preparation. a) Fabrication of FluidFaceMB and EV capture. Reproduced with permission.^[^ ^165^ ^]^ Copyright 2023, Wiley‐VCH, GmbH. b) Herringbone‐patterned microfluidic device for capturing HER2‐positive cancer exosomes in urine. Reproduced with permission.^[^ ^166^ ^]^ Copyright 2024, Elsevier. c) An immuno‐magnetophoresis‐based microfluidic chip for EV isolation. Reproduced with permission.^[^ ^167^ ^]^ Copyright 2023, Elsevier. d) EV isolation using a digital microfluidic platform. Reproduced with permission.^[^ ^168^ ^]^ Copyright 2024, Elsevier. e) Immuno‐inertial microfluidics for EV isolation. Reproduced with permission.^[^ ^169^ ^]^ Copyright 2023, Elsevier. f) A microfluidic device using metallic arrays for EV isolation. Reproduced with permission.^[^ ^170^ ^]^ Copyright 2023, Elsevier.

Advancements in microfluidic technologies have significantly impacted the efficiency of EV isolation. Initially, Zeng's group introduced a pioneering design by engineering a 3D‐nanopatterned microfluidic chip to improve the efficiency of EV isolation.^[^ ^163^ ^]^ This chip improved mass transfer, increased surface area, and optimized probe density, resulting in higher binding efficiency. Notably, its special nanopores facilitated boundary fluid drainage, minimizing hydrodynamic resistance and enhancing EV‐surface interactions. Building on this concept, Yang et al. enhanced the technology by developing a microfluidic chip with a nanoporous fluidic interface that features lipid bilayers conjugated with EpCAM aptamers.^[^ ^164^ ^]^ This design not only promoted molecular clustering at binding sites but also enhanced multivalent interactions, significantly improving capture efficiency. Wu et al. further extended their work by integrating fluid‐supported lipid bilayers on magnetic beads, creating a multivalent magnetic interface within a microfluidic system (**Figure** **4**a).^[^ ^165^ ^]^ This innovation accelerated biomolecular recognition kinetics, reduced nonspecific adsorption, and enabled efficient magnet‐assisted release of captured EVs. Expanding this paradigm, novel approaches aimed at enhancing the physical interaction between EVs and microfluidic structures emerged. Haam and colleagues introduced a chip featuring a 3D herringbone pattern, effectively capturing specific EVs by improving mass transfer through micro‐mixing (Figure 4b).^[^ ^166^ ^]^ This was achieved by stacking silica nanoparticles, which enhanced the contact and interaction between EVs and the structure. Using this nanochip to capture HER2‐positive EVs, the results confirmed a capture efficiency of ≈97.7%. Additionally, the same research group developed a system using magnetically labeled nanoclusters for targeted EV separation (Figure 4c).^[^ ^167^ ^]^ By synthesizing magnetic nanoclusters with varied magnetization and coating them with specific antibodies, they achieved effective separation through distinct migration trajectories within the microfluidic channels. The field saw further innovation with the application of digital microfluidics (DMF) technology, as demonstrated by Mao and collaborators (Figure 4d).^[^ ^168^ ^]^ Their DMF platform automates traditional EV isolation, enhancing time efficiency and reducing the total EV isolation duration to 20–30 min, with an EV isolation efficiency exceeding 77%. Besides, this platform integrates EV miRNA analysis for early disease screening, demonstrating its potential for rapid and efficient diagnostic applications. Warkiani et al. designed an ImmunoInertial microfluidics system, specifically aimed at isolating targeted subpopulations of small EVs (Figure 4e).^[^ ^169^ ^]^ The microfluidic chip's unique design features rigid zigzag microchannels, enhancing its effectiveness. This approach achieved an isolation efficiency of over 90%. Following isolation, the small EVs were meticulously characterized using flow cytometry and this platform also demonstrated the flexibility to process variable sample volumes. Recently, Hsiao et al. integrated polydimethylsiloxane microfluidic channels with metallic nanostructure arrays featuring tubular nano‐architectures to separate EVs derived from MCF7 cells (Figure 4f).^[^ ^170^ ^]^ A 95.3% recovery rate was achieved within one hour for a 500 µL EV sample. Concurrently, cyclic voltammetry operations effectively released the captured exosomes for downstream research applications. Each of these developments showcases the rapid evolution and diverse applications of microfluidic technologies in EV research. From the employment of magnetic interfaces and herringbone patterns to the utilization of digital platforms and metallic arrays, these methods represent the forefront of microfluidic technology in the pursuit of precise and efficient EV preparation.

#### 3.2.2 Latest Nanostructure‐Enabled Techniques

**Figure 5:**
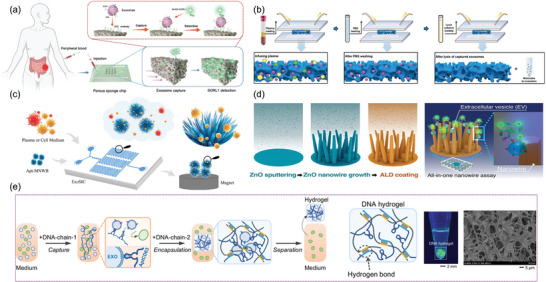
Nanomaterials, including nanoparticles and nanowires, serve as innovative tools for the efficient isolation of EVs. a) 3D porous sponge chip for EV isolation. Reproduced with permission.^[^ ^171^ ^]^ Copyright 2023, Wiley‐VCH, GmbH. b) SiO_2_ microsphere‐coated 3D hierarchical porous chip for the enrichment of EVs. Reproduced with permission.^[^ ^172^ ^]^ Copyright 2023, Wiley‐VCH, GmbH. c) ExoSIC chip for the continuous isolation of EVs. Reproduced with permission.^[^ ^173^ ^]^ Copyright 2023, Royal Society of Chemistry. d) ZnO nanowires for EV isolation. Reproduced with permission.^[^ ^176^ ^]^ Copyright 2023, American Chemical Society. e) DNA‐based hydrogel for EV isolation. Reproduced with permission.^[^ ^177^ ^]^ Copyright 2023, National Academy of Sciences.

Recent advancements in nanotechnology have significantly influenced the development of novel strategies for EV isolation, utilizing the unique properties of nanostructure, nanoparticles, and nanowires. Du et al. fabricated a microfluidic device incorporating porous nanostructured microarrays for capturing EVs (**Figure** **5**a).^[^ ^171^ ^]^ This chip, featuring a 3D porous sponge structure, enhances mass transfer in the microchannels, leading to capture efficiency approaching 90%. Similarly, Wang et al. engineered a 3D porous chip coated with SiO_2_ microspheres to enhance exosomes enrichment (Figure 5b).^[^ ^172^ ^]^ The chip's interconnected micropores create chaotic fluid flow, while the SiO_2_ microspheres decrease pore size, increasing surface area and reducing fluid boundary effects. This efficient method allows for direct exosomes isolation from plasma, achieving a detection limit of 10,000 particles per milliliter. Further advancing the field, Wu et al. engineered an irregular serpentine microfluidic chip (ExoSIC), which utilizes raspberry‐shaped magnetic nanobeads for the continuous isolation of EVs from plasma (Figure 5c).^[^ ^173^ ^]^ This chip features an irregular serpentine microchannel that enhances fluid mixing, significantly improving EV capture efficiency. The bespoke raspberry‐shaped nanobeads offer benefits beyond enhanced capture and exhibit size‐exclusion properties that improve EV purity. Subsequent work by Ye et al. presented a microfluidic chip with droplet‐shaped micropillar array structures in tandem with Tim4‐modified beads.^[^ ^174^ ^]^ This innovative design alleviates hydrodynamic resistance while augmenting fluid mixing, thus expediting sample processing and increasing capture efficiency. Advancements in nanowire technologies have also garnered attention for EV isolation. Thierry et al. employed iron oxide nanowires derived from biofilms to magnetically enrich immune‐specific EVs, achieving a yield of 83.7 ± 8.9%.^[^ ^175^ ^]^ The efficacy of their method draws from the notable surface area‐to‐volume ratio and swift magnetic responsiveness of the nanowires. In parallel, Baba et al. developed a nanowire‐based system for EV process, as shown in Figure 5d.^[^ ^176^ ^]^ Employing ZnO/Al_2_O_3_ core/shell nanowire, the design exhibited superior EV capture efficiency relative to uncoated ZnO nanowires, attributable to the augmented surface charge. The system's integration with traditional well‐plate detection techniques further streamlined the process, ensuring proficient capture and subsequent analysis of EVs. Furthermore, the rapid advancements in DNA technology have led to the development of highly selective and non‐destructive methods for EV preparation, notably a DNA‐based hydrogel that can be enzymatically cleaved to release isolated EVs (Figure 5e).^[^ ^177^ ^]^ In the study, a DNA hydrogel was engineered using ultra‐long single‐stranded DNA (ssDNA) derived through rolling circle amplification (RCA). The design of the RCA template incorporated an aptamer, termed AptCD63, specifically targeting CD63 present on exosomes. Owing to its design, this extended ssDNA exhibited a marked affinity toward CD63‐positive exosomes, facilitating their separation by the DNA hydrogel. These isolated exosomes have been successfully applied to the clinical diagnosis of human breast cancer and the treatment of myocardial infarction in rat models. Very recently, Li et al. proposed a novel method for the preparation of intact EVs featuring dual protein binding on the membrane. This method utilizes proximity ligation assay and DNA‐RNA hybridization, combined with functionalized magnetic beads, to selectively isolate EVs.^[^ ^178^ ^]^ These diverse methodologies highlight the intricate collaboration among material science, nanotechnology, and biochemistry in pursuing precise and effective techniques for the isolation of EVs. By leveraging the unique attributes of nanomaterials, from their structural innovations to their biochemical specificity, researchers are pushing the boundaries of what is achievable in EV preparation.

#### 3.2.3 Additional Cutting‐Edge Methods

Beyond the aforementioned techniques for EV preparation, physical parameters, such as dielectric properties, particle size and surface charge, play crucial roles in the development of innovative EV preparation strategies. Among these, acoustofluidics emerges as a pivotal technology. This method, a fusion of microfluidics and acoustics, employs acoustic waves to exert radiation forces on particles within microenvironment.^[^ ^179^ ^]^ Such forces drive particles toward acoustic nodes, with the radiation force being directly proportional to particle size. This proportionality allows for the precise separation of particles across a range of sizes.^[^ ^180^ ^]^ Remarkably, a novel acoustofluidic platform has been developed for label‐free and contact‐free EV isolation directly from whole blood, boasting an impressive yield exceeding 99% for particles as small as 110 nm.^[^ ^181^ ^]^ Dielectrophoresis offers another efficient methodology, exploiting the varying dielectric properties among EVs to induce inhomogeneous forces in a dielectrophoretic field.^[^ ^182^ ^]^ One such cutting‐edge insulator‐based dielectrophoresis device employs borosilicate micropipette arrays to isolate EVs from both cell culture media and biological fluids, achieving yields two orders of magnitude greater than traditional differential centrifugation methods, within 20 min and requiring only 200 µL of sample volume.^[^ ^183^ ^]^ Further advancements have seen the integration of electrothermal flow with dielectrophoresis into a single microfluidic platform. This platform features uniquely contoured 3D microelectrodes that generate electrothermal fluidic rolling. In conjunction with dielectrophoretic forces, this combined mechanism efficiently separates EVs from cell culture medium or serum, achieving yields of ≈80%.^[^ ^184^ ^]^ In addition to these methods, deterministic lateral displacement (DLD) method is also applied to separate particles exclusively based on size by utilizing gradient post arrays with specific geometries.^[^ ^185^ ^]^ One such system integrates a staggering 1024 nano‐DLD array structures on a single chip, capable of processing liquid samples at rates of up to 900 µL h^−1^.^[^ ^186^ ^]^ Another innovative technique utilizes viscoelastic fluids, a type of non‐Newtonian fluid that exhibits both viscosity and elasticity. These fluids are formulated by incorporating high‐molecular‐weight, water‐soluble polymers into either a buffer solution or the sample fluid itself.^[^ ^187^ ^]^ When synergistically combined with microfluidic technology, these viscoelastic media offer a promising avenue for the efficient separation and purification of EVs.^[^ ^188^ ^]^ A device developed by J. deMello et al., which leverages viscoelastic properties for microfluidic technology, showcases impressive efficiency in isolating small EVs from human blood, achieving 97% purity and 87% recovery rate.^[^ ^189^ ^]^ Another technology to be discussed involves the preparation of EVs using an externally applied magnetic field. This method leverages the principles of negative magnetophoresis, induced by applying an external magnetic field to a specialized magnetic solution. Within this environment, non‐magnetic particles experience a volume‐proportional repulsive force, thereby enabling efficient separation based on particle size.^[^ ^190^ ^]^ Recent innovations in this area have led to the development of a high‐gradient magnetic field module, which achieves label‐free EV separation with a recovery rate of 85.8% and a purity level of 80.45%.^[^ ^191^ ^]^

For a consolidated overview of the advanced EV preparation techniques discussed, the following table (**Table** **1**) outlines each method's sample type, targeted diseases, results, and distinguishing features. This structured presentation aims to provide a clear and comprehensive overview of the specifics of each technique.

**Table 1:** Overview of advanced techniques for EV preparation.

Method	Sample Types	Target Disease	Results	Specifics	Reference
3D‐Nanostructured microfluidic	Cell culture and urine	Breast cancer	Capture efficiency: ≈97.7%	Fluid mixing enhancement and boundary effect reduction	[166]
Immuno‐magnetophoresis‐based microfluidic	Cell culture and urine	NA	Separation efficiency: 90.9%	Magnetic nanocluster magnetization‐based EVs sorting	[167]
Digital microfluidic platform	Plasma and cell culture supernatant	Non‐small cell lung cancer	Isolation efficiency: 77%	Reusable chip and automated sample processing in 20–30 min	[168]
Immunoinertial microfluidic	Cell culture supernatant	NA	Efficiency: > 90%	High‐throughput for isolation of small EV subpopulations	[169]
Metallic nanostructure arrays microfluidic	Cell culture supernatant	NA	Recovery rate: 95.3%	EV release induced by cyclic voltammetry operations	[170]
3D porous sponge microfluidic chip	Cell culture supernatant and serum	Colorectal cancer	Capture efficiency: ≈90%	High surface‐to‐volume ratio, and enhance mass transfer	[171]
Microsphere‐coated 3D porous chip	Cell culture supernatant and plasma	Hepatocellular carcinoma	Detection limit of as low as 10 000 particles/mL^−1^	Large surface area and fluid boundary effects reduction	[172]
Magnetic‐nanowaxberry‐based microfluidic	Cell medium and plasma	Lung cancer	Recovery rate: > 83%	Irregular serpentine microchannels to increase fluid chaotic mixing	[173]
Nanowire‐based well plate	Organoid medium and glioblastoma patient urine	Glioblastoma	Captured particles: 3.75 × 10^6^ in 0.3 mL	A nanowire‐integrated system for EV capture through charge interactions	[176]
DNA nanotechnology	Serum	Human breast cancer	Separation efficiency: 8 × 10^7^ EVs per 50 µL	Nondestructively released	[177]
Acoustofluidics	Whole blood	NA	Purity: 98.4%	An integrated chip for EV isolation from undiluted whole blood	[181]
Electrothermal fluid /dielectrophoresis	Rabbit blood serum	NA	Recovery rate: 75.4 ± 3.3%	Device functional in high‐conductivity environment	[184]
Deterministic lateral displacement arrays	Serum and urine	Prostate cancer	Yield: ≈50%	High fluid throughput	[186]
Viscoelastic microfluidic	Human blood	Cancer	Recovery rate: 87%; Purity: 97%	Device combines dual functions in a single structure	[189]
Magnetic separation system	Cell culture supernatant	NA	Recovery rate: 85.8%; Purity: 80.45%	Ultra‐high gradient magnetic field module on‐chip	[191]

### 3.3 Comparison

The field of EV research presents a challenging landscape, primarily due to the heterogeneous nature and diverse origins of EVs.^[^ ^58e^ ^]^ This diversity poses significant obstacles to achieving consistent outcomes, a critical step for downstream analyses and applications. Despite the emergence of diverse preparation techniques (summarized in **Table** **2**), the absence of standardization remains, hindering the consistency of results. Inconsistencies arise both from variations in the outcomes of different methods applied to the same sample and from a single method yielding different results across samples.^[^ ^51^, ^103^, ^192^ ^]^ To address these challenges, several studies have been dedicated to assessing the impact of specific preparation methods on EV recovery rates, particle size distribution, and proteomic profiles. One investigation focused on four widely used techniques: ExoQuick™, the Total Isolation Kit, UC, and UF. The study provided evidence for substantial variations across these methods regarding particle concentration, size distribution, and protein content, underscoring the absence of a one‐size‐fits‐all approach.^[^ ^193^ ^]^ In a similar vein, another line of research assessed the effectiveness of three distinct methods‐UC, UC‐SEC, and UC‐DG‐for isolating EVs from pleural effusions. This research indicated that the UC‐SEC method yielded the purest EVs and the highest protein content, marking it as a favorable technique in specific settings.^[^ ^194^ ^]^ Further insights into the subject are provided by a study from Ikezu et al., which applied additional methods like SEC, Phosphatidylserine Affinity Capture, and Sucrose Gradient Ultracentrifugation (SG‐UC) to prepare EVs from the temporal lobe cortex of elderly individuals.^[^ ^195^ ^]^ Comprehensive assessments, leveraging TEM and NTA, attested to SG‐UC's superior recovery rates and effective enrichment of common EV proteins with minimal non‐EV proteins contamination. Concomitantly, existing research underscores the pivotal role that the selection of an EV preparation technique plays in influencing downstream analytical processes, such as proteomic and miRNA sequencing assays.^[^ ^196^ ^]^ From the research mentioned above, it becomes evident that the choice of EV preparation method significantly influences EV characterization and molecular profiling, which impacts downstream applications. Given these findings, it is paramount for researchers in the EV field to adhere to standardized guidelines, such as the MISEV2018/2023 guidelines, maintain meticulous experimental records, and establish appropriate controls. This approach will enhance the reliability and repeatability of results, thereby advancing the understanding and application of EVs.

**Table 2:** The strengths and weakness of various purification approaches.

Approaches	Strengths	Weaknesses
Ultracentrifugation	Pervasively adopted Relatively elevated levels of purity	Human resource‐demanding Incapable of facile scalability High costs for machinery Low yield
Density Gradient Centrifugation	High purity Compatible with diverse, well‐prepared biological samples	Human resource‐intensive Requiring extended time allocation Incapable of facile scalability Low yield
Size Exclusion Chromatography	Universally employed across various applications Effectively remove the contaminant proteins Commercially columns accessible	Risk of co‐isolating size‐similar contaminants Low‐concentration EVs necessitate further concentration
Ultrafiltration	Facile scalability High yield Common for initial clean‐up or post‐isolation concentration	Suboptimal purity Pressure‐induced EV damage Filtration clogs in large volumes
Precipitation	Fast processing Low‐cost method High yield	Low purity Non‐specific Binding
Asymmetrical Flow Field‐Flow Fractionation	Rapid and efficient High‐yield potential Easily scalable	Necessitates high‐cost instrumentation Demand comprehensive procedural fine‐tuning
Tangential Flow Filtration	High Throughput Scalability Minimal Sample Damage	Equipment cost Risk of pore blocking Possible Contaminant Co‐isolation
Anion Exchange Chromatography	Low Mechanical Stress Reproducibility No Special Equipment	Limited suitability for complex biofluids Optimization Required
Immunoaffinity	High‐purity EVs Targeted EV subtype isolation No special equipment	Limited scalability Elution difficulty affecting exosomes integrity Antibodies employed for EVs capture lack clinical validation
Microfluidic Platform	Concurrent isolation and profiling Low‐volume samples Enhanced purity in multi‐step protocols	Validation and Standardization High device development costs Scalability challenges

## 4 EV Analysis Techniques

The analysis of EVs poses a unique set of challenges, primarily due to their minuscule size and the notably low concentrations of bioactive components they contain, including proteins, nucleic acids, lipids and metabolites.^[^ ^197^ ^]^ These difficulties are further compounded by the inherent heterogeneity and diverse origins of EVs, which stem from variations in their cellular origins, the physiological state of the source cells, and the mechanisms through which they are secreted.^[^ ^198^ ^]^ Consequently, the task of achieving accurate and comprehensive profiling of EVs is not only challenging but also emerges as a significant obstacle to leveraging their potential in clinical settings.^[^ ^199^ ^]^

**Figure 6:**
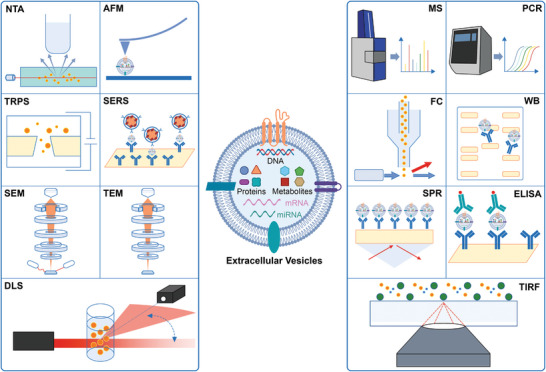
Various methods for EV analysis include: **NTA**: Nanoparticle Tracking Analysis; **AFM**: Atomic Force Microscopy; **TRPS**: Tunable Resistive Pulse Sensing; **SERS**: Surface‐Enhanced Raman Scattering; **SEM**: Scanning Electron Microscopy; **TEM**: Transmission Electron Microscopy; **DLS**: Dynamic Light Scattering; **MS**: Mass Spectrometry; **PCR**: Polymerase Chain Reaction; **FC**: Flow Cytometry; **WB**: Western Blotting; **SPR**: Surface Plasmon Resonance; **ELISA**: Enzyme‐Linked Immunosorbent Assay; **TIRF**: Total Internal Reflection Fluorescence.

The analysis of EVs encompasses a range of methodologies designed to elucidate their biophysical and biochemical attributes. Biophysical properties, such as size, morphology, surface charge, and particle concentration, provide foundational insights into EVs' physical nature. Concurrently, the analysis of biochemical features, including specific proteins, nucleic acids, and other cargos, reveals the functional and molecular complexity of EVs. To aid in the understanding of these diverse characterization methods, **Figure** **6** offers a visual overview, while **Table** **3** details their respective advantages and potential limitations. Building upon this foundation, the next section delves into the methodologies employed in EV analysis, with a focus on state‐of‐the‐art techniques. It also encompasses an exploration of advancements in single EV research, illustrating the field's continuous evolution and the innovative approaches shaping the future of EV research.

**Table 3:** The summary of EV analysis methods.

Methods	Benefits	Potential Drawbacks	Reference
Transmission Electron Microscopy	High‐resolution Minimal sample volume needed	Low throughput Sample preparation: laborious, risk of artifacts	[37, 200]
Scanning Electron Microscopy	Simpler sample preparation: less complex than TEM Minimal sample volume needed	Lower resolution: compared to TEM Risk of artifacts from sample dehydration. Might obscure/alter sample by conductive coating	[201]
Atomic Force Microscopy	Can provide 3D surface topography Probe the mechanical characteristics Analysis in physiological environments	Low throughput Expertise needed: complex use and data interpretation	[40, 202]
Nanoparticle Tracking Analysis	Provide both size distribution and particle concentration Real‐time visualization Better resolution for polydisperse samples	Difficulty detecting particles smaller than ≈30 nm More complex sample preparation	[203]
Dynamic Light Scattering	Require minimal steps before analysis Rapid determination of size distribution	No concentration data. Polydisperse challenge: difficult to accurately determine size in samples with a broad size distribution	[36, 204]
Tunable Resistive Pulse Sensing	Provide particle size and concentration Adjustable for different particle sizes	Particles or aggregates can block the pore Sensitive to changes in temperature, salt concentration, and other factors	[41, 205]
Western Blotting	Confirm the presence of specific EVs markers Can probe multiple proteins using different antibodies	Time‐consuming Not ideal for low‐abundance proteins	[42, 206]
Enzyme‐Linked Immunosorbent Assay	Provide quantitative data on protein concentrations Suitable for analyzing multiple samples simultaneously	Chance of antibodies detecting unintended targets Might not detect very low abundance proteins	[43, 207]
Mass Spectrometry	Broad profiling of proteins, lipids, and other molecules Can detect molecules present in low concentrations	Require advanced bioinformatics tools Need for rigorous sample preparation High setup and maintenance costs	[44, 208]
Polymerase Chain Reaction	Can detect low quantities of specific nucleic acid sequences Provide quantitative data	Sensitive to the purity of EV nucleic acid Risk of sample contamination leading to false positives	[45, 209]
Total Internal Reflection Fluorescence	High signal‐to‐noise ratio Enable real‐time observation of EVs interactions at the cell membrane or surface	Limited field of view Require precise setup alignment and calibration	[210]
Flow Cytometry	Can simultaneously measure multiple parameters (e.g., size and protein markers) Rapid data acquisition Provide information on individual EVs	Detection of smaller EVs can be challenging Non‐specific binding Require instruments with high sensitivity and resolution	[211]
Surface Plasmon Resonance	Capable of detecting low concentrations Label‐free detection	Highly sensitive to changes in refractive index Require careful preparation and optimization of the sensor chip surface	[46, 212]
Surface‐Enhanced Raman Scattering	Detect low‐abundance molecules Provide multiple targets simultaneously based on distinct Raman shifts	Reproducibility not ideal Background interference	[47, 213]

### 4.1 Biophysical Analysis

The multifaceted characterization of EVs benefits from an array of advanced microscopy techniques, each offering unique insights into their morphology. Transmission Electron Microscopy (TEM) excels in delineating the ultrastructure of EVs at high resolution. Operating under vacuum conditions, TEM employs accelerated electron beams to penetrate the sample, subsequently forming an image on a screen based on the scattered and transmitted electrons. The technique relies on differences in electron‐scattering properties of the materials within the sample to produce contrast, with denser materials appearing darker.^[^ ^200^ ^]^ This property is especially useful for highlighting the lipid membranes of EVs through negative staining with heavy metal salts like osmium tetroxide and uranyl acetate.^[^ ^214^ ^]^ For targeted protein analysis on the EV surface, immuno‐gold labeling techniques are deployed.^[^ ^37^, ^215^ ^]^ Cryo‐Electron Microscopy (Cryo‐EM) addresses potential morphological distortions introduced during sample preparation in traditional microscopy. This technique involves the rapid freezing of liquid samples, permitting their observation under low‐temperature conditions. Especially, Cryo‐EM imagery predominantly portrays EVs as spherical entities.^[^ ^39^, ^216^ ^]^ Scanning Electron Microscopy (SEM) focuses on surface morphology by utilizing secondary electron signals. A fine‐focused electron beam scans the sample surface, inducing the emission of secondary electrons which are subsequently collected by a specialized detector. The resultant electrical signals generate an image on a screen.^[^ ^201^, ^217^ ^]^ It's worth noting that SEM operates in a vacuum and typically necessitates sample preparation steps like adsorption fixation and dehydration.^[^ ^201a^ ^]^ Such treatments often give rise to EV images characterized as butterfly or saucer‐shaped.^[^ ^201^, ^218^ ^]^ Atomic Force Microscopy (AFM), also known as Scanning Force Microscopy, enriches the array of available techniques with its capability to image EVs at nanoscale resolution under various environmental conditions, including atmospheric, vacuum, or liquid settings.^[^ ^219^ ^]^ In AFM, a mechanical cantilever scans the EV surface, with its deflection modulating in response to surface topology. This method requires the adsorption of EVs onto smooth substrates like mica or treated glass slides, which are then gently dried.^[^ ^220^ ^]^ Subsequently, a variety of probes and operational modes can be employed for the intricate scanning and imaging of EVs.^[^ ^220^, ^221^ ^]^

Expanding upon the exploration of morphology‐centric techniques for EV characterization, the discourse now shifts to methods that evaluate particle size distribution, surface charge, and concentration. Three techniques, namely Nanoparticle Tracking Analysis (NTA), Dynamic Light Scattering (DLS), and Tunable Resistive Pulse Sensing (TRPS), stand at the forefront of this domain, each contributing uniquely to the analysis of EVs. NTA, a widely embraced technique, simultaneously quantifies the size distribution and concentration of EVs in suspension.^[^ ^35^, ^222^ ^]^ By leveraging light scattering and Brownian motion, NTA shines a laser beam on EVs through a glass prism and collects the scattered light with an optical microscope to track the particles' motion. This method provides detailed insights across a broad spectrum of EV sizes, requiring meticulous calibration and parameter tuning for accurate data collection. Besides size and concentration, NTA's capabilities include fluorescence and surface charge detection, enhancing its versatility in EV analysis. DLS, also known as quasi‐elastic light scattering, complements NTA by offering non‐invasive measurements of particle size and distribution, particularly within the submicron range.^[^ ^36^, ^204^ ^]^ It analyzes the temporal fluctuations in scattered light intensity caused by the Brownian motion of suspended particles. This velocity is then used to deduce the particle size through the Stokes‐Einstein equation. While DLS is adept at determining particle size and surface charge, it does not provide concentration data. TRPS, a rapid, nanopore‐based technique, enables single‐particle level analysis of both size distribution and concentration.^[^ ^41^, ^205^, ^223^ ^]^ It works by detecting transient changes in ionic current as nanoparticles pass through a nanopore, with the magnitude of these changes indicating particle size. TRPS measures both size distribution and surface charge with precision, demonstrating its value in nanoparticle characterization.

### 4.2 Biochemical Analysis

The study of EVs is pivotal for understanding their roles in cellular communication and their potential in diagnostic and therapeutic applications. The importance of this is rooted in the wide array of bioactive signaling molecules within EVs, including proteins and nucleic acids. Consequently, a detailed biochemical examination of these molecules in EVs is crucial. Thus, a thorough biochemical characterization of these molecules within EVs is essential. It not only deepens our understanding of EVs' functions but also uncovers their clinical applications. Here, we present a critical overview of both established and emerging techniques in the biochemical analysis of EVs, highlighting the most cutting‐edge methods.

Western Blotting (WB) is fundamental in the biochemical sciences for profiling EV proteins. The process involves treating purified EVs with a buffer containing denaturants and protease inhibitors, followed by protein separation through sodium dodecyl sulfate‐polyacrylamide gel electrophoresis. The proteins are then transferred to a cellulose membrane for detection using specific antibodies and enhanced chemiluminescence.^[^ ^42^, ^206^ ^]^ Established protocols for EV protein WB exist, as illustrated by the work of Kahn et al. In their study, they conducted an extensive analysis of EV proteins from various cell lines and observed differential abundances of tetraspanins.^[^ ^224^ ^]^

Enzyme‐Linked Immunosorbent Assay (ELISA), a cornerstone for quantifying proteins, extends its utility to EV research with specialized kits. The sandwich ELISA format allows for the quantification of EV proteins by capturing EVs or their lysates on a solid‐phase carrier, followed by detection with a secondary antibody.^[^ ^43^, ^207^ ^]^ A recent advancement in this domain involves the utilization of thiolated‐nicotinamide adenine dinucleotide cycling, a method that has enabled ultra‐sensitive detection of the EVs‐specific protein GRP78.^[^ ^207a^ ^]^ Further enriching the methodological landscape, a high‐throughput droplet digital ELISA has been introduced, boasting the capability to generate 20 million droplets per minute and a detection sensitivity reaching as low as 9 EVs µL^−1^.^[^ ^225^ ^]^

Mass Spectrometry (MS) is a powerful technique for analyzing complex samples by ionizing compounds and sorting them based on their mass‐to‐charge ratio. It has been instrumental in the proteomic analysis of EVs, with advances in chromatography‐coupled MS enhancing the identification of new EV protein biomarkers.^[^ ^44^, ^226^ ^]^ The proteomic study of EVs typically involves a three‐step process: isolation and purification of EVs, identification of proteins through MS, and detailed data analysis. Noteworthy methodological developments include an in‐situ signal amplification technique using Matrix‐Assisted Laser Desorption Ionization‐Time of Flight MS (MALDI‐TOF MS) for quantifying GPC1(+) EVs.^[^ ^227^ ^]^ Another study combined inductively coupled plasma mass spectrometry (ICP‐MS) with nanoparticle liquid biopsy for pancreatic cancer‐derived EVs.^[^ ^228^ ^]^ This method's specificity and sensitivity have made significant strides in EV research, complemented by efforts to characterize EV lipids and metabolites, marking progress in the fields of EV lipidomics and metabolomics.^[^ ^86^, ^229^ ^]^

Polymerase Chain Reaction (PCR) is widely acknowledged for its ability to detect nucleic acids, including the analysis of the varied nucleic acid compositions found within EVs. Initial research, utilizing techniques such as RT‐qPCR and microarray, verified the presence of various RNA types, including mRNA, miRNA, and lncRNA, within EVs.^[^ ^209^, ^230^ ^]^ The advent of high‐throughput RNA sequencing has considerably broadened our understanding, revealing a wide range of RNA species in EVs, including not only the previously mentioned RNA types but also circRNA, piwi‐interacting RNA (piRNA), ribosomal RNA (rRNA), YRNA, transfer RNA (tRNA), small nuclear RNA (snRNA), and small nucleolar RNA (snoRNA).^[^ ^231^ ^]^ These RNA molecules (mainly mRNAs, microRNAs, circRNAs, and lncRNAs) are of great interest because of their important roles in cellular communication, disease pathways, and as potential biomarkers and therapeutic targets.^[^ ^80^, ^81^, ^232^ ^]^ Specialized databases, such as exoRBase and EVmiRNA, developed from public RNA‐seq data, facilitate deeper exploration of exosomal molecules and their applications.^[^ ^233^ ^]^ For RNA extraction from EVs, techniques such as phenol‐chloroform and column centrifugation have been standardized and are available commercially in kit forms.^[^ ^234^ ^]^ Following extraction, conventional methods like quantitative RT‐PCR or RNA‐Sequencing are typically employed to analyze the transcriptomics of EV RNAs.^[^ ^45^, ^209^ ^]^ Steven et al. utilized RT‐ddPCR and Nanostring nCounter for EV's mRNA analysis to shed light on breast cancer's molecular subtypes.^[^ ^235^ ^]^ Song et al.’s work focused on the role of exosomal circRNAs in colorectal cancer, employing qRT‐PCR and analyzing the circRNA‐miRNA‐mRNA network for insights into their functionality and clinical relevance.^[^ ^236^ ^]^ Zhai et al. undertook exosomal RNA sequencing to discover that, in early esophageal squamous cell carcinoma, exosomal lncRNAs displayed more pronounced tissue specificity and higher expression levels with reduced splicing efficiency compared to mRNAs.^[^ ^237^ ^]^ Beyond these traditional methods, various biosensor platforms and microfluidic devices are utilized to analyze EV RNA, enhancing their feasibility for clinical settings and point‐of‐care testing applications.^[^ ^232a^ ^]^ Due to the generally low concentration of nucleic acids in EVs, signal amplification techniques such as strand displacement reaction (SDR),^[^ ^238^ ^]^ hybridization chain reaction (HCR),^[^ ^209^, ^239^ ^]^ rolling circle amplification (RCA),^[^ ^240^ ^]^ CRISPR/Cas,^[^ ^201^, ^241^ ^]^ and catalytic hairpin assembly (CHA)^[^ ^242^ ^]^ are frequently utilized to improve detection sensitivity. One recent study serves as an example. Jiang et al. reported a method that simultaneously detects surface proteins and miRNAs of EVs using microfluidic channels modified with CD63 aptamers.^[^ ^243^ ^]^ This method achieved a detection limit of 83 EVs per microliter through the use of Cy5‐labeled specific aptamers and a CHA‐based signal amplification strategy.

Total Internal Reflection Fluorescence (TIRF) analysis represents a sophisticated approach for observing single molecules or nanoparticles by monitoring their fluorescence following total internal reflection excitation. This method facilitates accurate quantification by counting fluorescence spots and measuring their intensity.^[^ ^210^, ^244^ ^]^ Leveraging TIRF, a key study established a high‐throughput nano‐biochip system for liquid biopsy applications, allowing simultaneous detection of proteins and mRNA in EVs.^[^ ^245^ ^]^ In this system, specific antibodies targeting CD9, CD63, and PD‐L1 were tagged with fluorescent markers for protein identification, while molecular beacons were employed to detect mRNA, involving the integration of EVs with beacon‐equipped lipid vesicles. This innovative system is notable for its minimal sample requirements, rapid detection, and high‐throughput capabilities. Extending this research, Li and colleagues introduced a TIRF‐based technique for in situ quantification of miRNA in tumor‐derived EVs.^[^ ^210a^ ^]^ This detection scheme involves the incorporation of fluorescence‐quenched substrates into exonuclease O‐treated EVs to initiate a target miRNA‐activated catalytic cleavage reaction, thereby amplifying fluorescence signals.

Flow cytometry (FC) is an advanced technique for rapidly and quantitatively analyzing cells or cell‐sized particles in suspension.^[^ ^246^ ^]^ Unlike preceding methods of EV characterization, which are often limited to examining individual attributes, flow cytometry offers integrated multi‐parametric analysis that includes both physical and biochemical characteristics of EVs. Light scattering techniques are used to determine particle size, while multi‐color fluorescent markers facilitate the detection of intracellular gene expression, specific protein expression, and particle concentrations.^[^ ^247^ ^]^ However, traditional flow cytometers face sensitivity limitations, which make it challenging to detect EVs smaller than 300–500 nm.^[^ ^248^ ^]^ To address this challenge, two main strategies have emerged. The first involves artificially enlarging the size of the EVs by binding them to magnetic or latex beads, enabling detection on conventional flow cytometry systems.^[^ ^211^, ^249^ ^]^ Alternatively, the advent of high‐sensitivity nano‐flow cytometry technology, as demonstrated by devices like the Apogee A50 and NanoFCM, to analyze smaller particles directly.^[^ ^250^ ^]^ Uniquely, NanoFCM allows for the quantitative multi‐parametric analysis of individual particles with sizes as small as 40 nm.^[^ ^250c^ ^]^ To ensure consistency and standardization in the flow cytometric analysis of EVs, comprehensive guidelines have been established, embodied in the Minimum Information about a Flow Cytometry Experiment (MIFlowCyt) standard, adapted to include an EV‐specific reporting framework, MIFlowCyt‐EV.^[^ ^211c^ ^]^

Surface Plasmon Resonance (SPR) is an optical biosensing technique for real‐time, label‐free assessment of material properties by measuring refractive index changes in proximity to a metal surface.^[^ ^212b^ ^]^ With its wide‐ranging applications in pharmaceuticals, genetics, and protein analysis, SPR has recently been extended to characterize EVs.^[^ ^46^, ^212^, ^251^ ^]^ In the realm of EV research, SPR technology leverages capturing agents such as antibodies, aptamers, or peptides, which are immobilized on metallic substrates. This setup is designed to secure target EVs, with their binding leading to localized refractive index changes. These changes are meticulously tracked through optical signal variations, enabling detailed analyses.^[^ ^212a^ ^]^ Studies have reported the quantitative analysis of transmembrane proteins CD9, CD63, and PD‐L1 in EVs using SPR technology.^[^ ^251^, ^252^ ^]^ One notable study employed a commercial Protein A SPR sensor, devoid of additional modifications, to directly assess the presence of transmembrane proteins in EVs extracted from human lung cancer cells, achieving a detection sensitivity within the range of (0.6 − 1.8) × 10^4^ particles mL^−1^.^[^ ^253^ ^]^ Expanding upon these technological advancements, Wu et al. introduced an integrated biosensor for the concurrent detection of both protein and miRNA content in tumor‐derived EVs.^[^ ^254^ ^]^ In their approach, antibodies were first immobilized on gold‐coated substrates to selectively capture target EVs. SPR was then deployed to quantify the EV proteins by detecting induced changes in the refractive index. Next, molecular beacons were employed to evaluate the miRNA profiles of the captured EVs, thereby facilitating a comprehensive molecular characterization.

**Figure 7:**
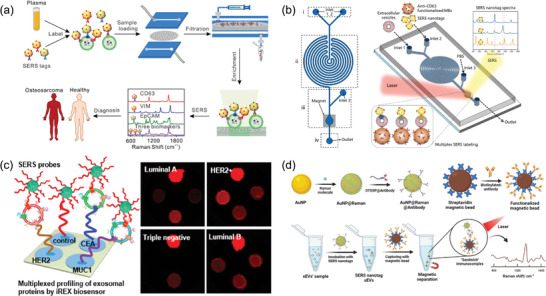
Labeled SERS for analysis of EVs. a) SERS profiling of three biomarkers on plasma‐derived exosomes for the diagnosis of osteosarcoma. Reproduced with permission.^[^ ^257^ ^]^ Copyright 2022, Elsevier. b) Structure and working principle of the on‐chip SERS‐based EV analysis platform. Reproduced with permission.^[^ ^258^ ^]^ Copyright 2023, Elsevier. c) A paper‐based SERS‐vertical flow biosensor for multiplexed quantitative profiling of EV proteins. Reproduced with permission.^[^ ^259^ ^]^ Copyright 2023, American Chemical Society. d) The “sandwich” SERS immunoassay for small EVs. Reproduced with permission.^[^ ^260^ ^]^ Copyright 2023, Royal Society of Chemistry.

Surface‐Enhanced Raman Scattering (SERS) significantly amplifies Raman scattering intensity for molecules attached to roughened metal surfaces such as silver, gold, and copper, making it a powerful tool for high‐sensitivity, non‐destructive analysis.^[^ ^213^, ^255^ ^]^ Consequently, its adoption has become extensive in the biomedical domain, especially in identifying EVs. SERS utilizes two primary approaches for EV detection: labeled and label‐free methods.^[^ ^47^, ^256^ ^]^ The labeled method enhances detection sensitivity and specificity by attaching Raman probe molecules to EVs and quantifying the resulting molecular intensities. In contrast, the label‐free method detects the intrinsic Raman signals of EVs, frequently utilizing artificial intelligence to process the raw data. Recent advancements highlight the innovative application of SERS in EV research. One study used an integrated microfluidics‐SERS method that employed tangential flow filtration to isolate EV immunocomplexes for analysis, achieving a detection limit as low as two EVs per microliter in blood plasma (**Figure** **7**a).^[^ ^257^ ^]^ Another utilized a serpentine microstructure chip combined with SERS to allow for the hands‐free detection of three tumor‐associated biomarkers, with detection limits ranging from 446 to 5460 particles per milliliter (Figure 7b).^[^ ^258^ ^]^ Further advancements were made by Li et al., who introduced a paper‐based SERS‐vertical flow biosensor for the multiplexed quantitative profiling of serological EV proteins (Figure 7c).^[^ ^259^ ^]^ This biosensor's vertical flow design minimizes false negatives and allows for the detection of multiple EV proteins on a single membrane, enhancing the efficiency of protein profiling. While various “sandwich” immunocomplex assembly techniques have been introduced in initial SERS‐based EVs research, comparative efficacy studies were lacking until a recent investigation explored three different methodologies. This study found that pre‐mixing small EVs with SERS labels before analysis produced the strongest signals, with a detection limit of 1.5×10^5^ particles per µL (Figure 7d).^[^ ^260^ ^]^ Additionally, a novel application of Hollow Core Anti‐Resonant Fibers (HcARF) in Raman spectroscopy has shown promise for EV protein detection.^[^ ^261^ ^]^ Compared to conventional flat substrates, HcARF elevated Raman signal strengths by three orders of magnitude. When integrated with SERS, this resulted in dual signal amplification, facilitating the concurrent detection of multiple proteins of EVs sourced from several cell lines. The advancement of labeled SERS in EV detection highlights increasing precision and adaptability. By employing molecular tags, these methods achieve exceptional specificity. Coupled with advanced microfluidic platforms, they offer deep insights into EV molecular composition, significantly enhancing diagnostic and research capabilities in biomedicine.

**Figure 8:**
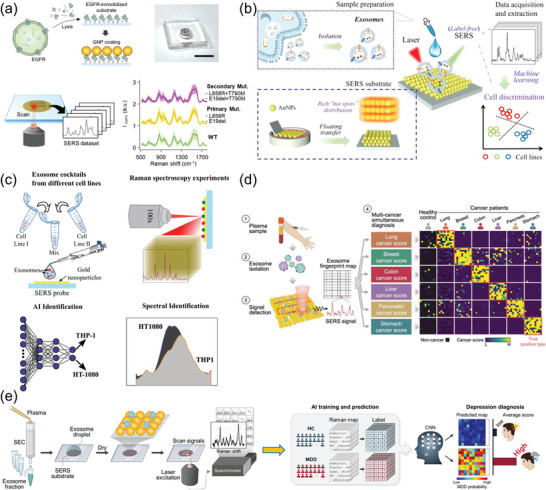
Recent progress in AI‐assisted SERS for the analysis of EVs. a) SERS dataset with deep‐learning algorithm for the analysis of plasma exosomal proteins. Reproduced with permission.^[^ ^265^ ^]^ Copyright 2023, American Chemical Society. b) Label‐free SERS combined with machine learning for EV detection. Reproduced with permission.^[^ ^266^ ^]^ Copyright 2023, American Chemical Society. c) Cellular origin identified by AI‐assisted SERS of EV analysis. Reproduced with permission.^[^ ^267^ ^]^ Copyright 2023, Wiley‐VCH, GmbH. d) Multi‐cancer simultaneous diagnosis utilizing AI‐SERS in EV analysis. Reproduced with permission.^[^ ^268^ ^]^ Copyright 2023, Springer Nature. e) Diagnosing major depressive disorder (MDD) in vitro through the detection of plasma exosomes via SERS and analysis using AI. Reproduced with permission.^[^ ^269^ ^]^ Copyright 2023, American Chemical Society.

Compared to label‐based SERS, label‐free SERS presents the distinct advantage of eliminating the requirement for elaborate antigen‐antibody labelling and this approach directly facilitates the capture of Raman spectra.^[^ ^213^, ^262^ ^]^ This approach streamlines the workflow and reduces financial and procedural burdens associated with labeling processes. Nonetheless, this method presents certain challenges, particularly due to the pronounced heterogeneity of EV samples that can yield intricate signals that are challenging to decipher.^[^ ^263^ ^]^ In response, researchers have turned to sophisticated analytical tools, combining multivariate statistical techniques with deep learning algorithms. This combination has proven effective in analyzing SERS signals, leading to the development of discriminative models that can identify EVs from various biological sources with high accuracy.^[^ ^264^ ^]^ For instance, one study demonstrated the use of Raman spectroscopy integrated with deep learning to identify protein mutations in plasma EVs from lung cancer patients (**Figure** **8**a).^[^ ^265^ ^]^ The process began with acquiring Raman spectra of EV proteins through SERS, followed by the application of a deep learning algorithm to discern unique protein features. Another study employed machine learning‐assisted SERS to analyze serum EVs, achieving high diagnostic sensitivity for detecting breast and cervical cancers (Figure 8b).^[^ ^266^ ^]^ By using a 3D ordered array of gold nanoparticles as the SERS substrate and combining principal component analysis with linear discriminant analysis, researchers could distinguish between EVs from normal and cancer cells, including different cancer cell lines. Beyond cancer detection, ongoing research has explored tracing the cellular origins of heterogeneous EV populations. For example, a study used a combination of SERS and machine learning to categorize EVs from five different cellular lines (Figure 8c).^[^ ^267^ ^]^ This computational strategy revealed a correlation between the predictive accuracy of label‐free Raman methodologies and the proportion of EVs derived from specific cell present within the examined samples. Further expanding the applications of this technology, a study has unveiled a method combining artificial intelligence and SERS for concurrently diagnosing multiple cancer subtypes via the analysis of label‐free plasma EVs (Figure 8d).^[^ ^268^ ^]^ This dual‐algorithmic strategy first classified each signal as normal or malignant, assigning a probability score. Then, a multi‐classifier model extracted tissue origin details for positively predicted results, providing insights into the tissue origins of oncogenic plasma EVs. In another study, researchers employed artificial intelligence to diagnose Major Depressive Disorder by analyzing the Raman spectral characteristics of plasma EVs (Figure 8e).^[^ ^269^ ^]^ Using a dataset of 28 000 SERS signals from EVs, a predictive deep learning model demonstrated an Area Under the Curve (AUC) of 0.939, with a sensitivity of 91.4% and a specificity of 88.6%. Through these examples, it is evident that label‐free SERS, supported by advanced statistical and machine learning techniques, represents a powerful tool for the non‐invasive diagnosis of various diseases, including cancer and Major Depressive Disorder. This approach offers not only a cost‐effective alternative to traditional methods but also holds promise for unraveling the complex heterogeneity and potential of EVs.

In further exploring advanced technologies for EV analysis beyond the previously mentioned integration of AI with SERS, various computational methods have emerged as indispensable in EV research. For instance, Huang et al. developed a computational strategy to illustrate the relative and absolute enrichment of tissue and cell types in plasma EVs, based on long RNA sequencing data from these vesicles.^[^ ^270^ ^]^ Similarly, Davis et al. presented a computational study of serum‐derived EV miRNAs in a juvenile sheep Fontan procedure model. The analysis revealed that Fontan‐overexpressed EV miRNAs target liver cells, highlighting a key pathway in Fontan‐related liver dysfunction.^[^ ^271^ ^]^ As the field of EV research continues to evolve, several key computational techniques have risen to prominence, laying the foundation for innovative analyses and discoveries. Techniques such as Linear Discriminant Analysis (LDA), Partial Least Squares Discriminant Analysis (PLS‐DA), Principal Component Analysis (PCA), Support Vector Machines (SVM), k‐nearest neighbors (KNN), XGBoost, and a variety of deep learning approaches‐including convolutional neural networks (CNNs), residual neural networks (ResNets), and artificial neural networks (ANNs)‐are at the forefront of this field.^[^ ^272^ ^]^ These methods, individually or in combination (like PCA‐LDA), have proven effective across a range of EV analysis, such as proteomics,^[^ ^273^ ^]^ metabolomics,^[^ ^274^ ^]^ transcriptomics,^[^ ^275^ ^]^ and disease diagnosis.^[^ ^276^ ^]^ The application of these computational strategies has significantly broadened our comprehension of the molecular intricacies of EVs, simultaneously paving the way for advancements in biomedical applications. Below, we will summarize the strengths and weaknesses of these computational methods, providing a concise overview alongside applications in EV analysis, as detailed in **Table** **4**.

**Table 4:** The summary of computational methods/algorithms for EV analysis.

Methods/ Algorithms	Strengths	Weaknesses	Applications in EV analysis	Reference
PCA	Preserve original data and maximize eigenvalues	Challenging to analyze minor variations	EV transcriptomics/proteomics	[273, 277]
LDA	Optimize class distinction and surpass PCA in classification	Errors and overfitting risk with small sample size	Exosomal multi‐miRNA analysis	[278]
KNN/SVM	Effectiveness in high dimensional spaces and simplicity	Inefficient with missing data and costly for large datasets	Correlating urinary EV biomarkers signals with clinical states	[279]
XGBoost	Efficiency and scalability	Model complexity and time‐consuming	Identification of EVs from different sources	[280]
PLS‐DA	Effectiveness with small sample sizes	Model complexity and overfitting risk	Identifying candidate biomarkers in EV metabolomics	[274, 281]
PCA‐LDA	Improved classification accuracy and overcoming overfitting	Sensitivity to data scaling	Distinguishing cancerous from normal exosomes subtype	[282]
ResNet	Boosts training stability and generalization accuracy	Require more computational resources and optimization difficulty	Predicting lung cancer from cell and plasma exosomes correlation	[263, 283]
ANN	Effectively handle large datasets and nonlinear data	Require large amounts of data resources	Classifying breast cancer subtypes via EV SERS spectra	[264, 284]
CNN	High specificity and sensitivity	Requires substantial computational resources	Diagnosing MDD with Plasma EV Raman features	[269, 284]

**Figure 9:**
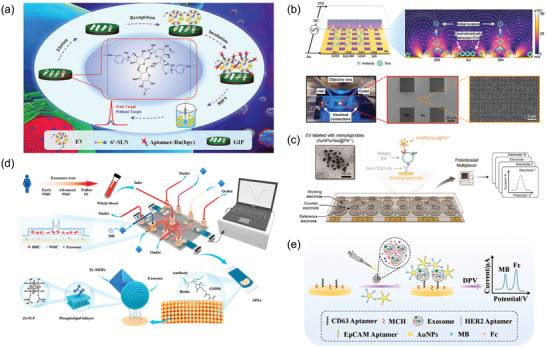
Recent advances in electrochemical sensors for EV analysis. a) Electrochemiluminescence for EV detection utilizing a glycosyl‐imprinted polymer membrane and an aptamer for dual recognition. Reproduced with permission.^[^ ^287^ ^]^ Copyright 2024, American Chemical Society. b) Enhanced electrokinetic, label‐free plasmonic sensing for the detection of tumor‐derived EVs. Reproduced with permission.^[^ ^288^ ^]^ Copyright 2023, Elsevier. c) Detection of urinary EVs using an electrochemical immunoassay in a microtiter plate. Reproduced with permission.^[^ ^289^ ^]^ Copyright 2023, American Chemical Society. d) The filter‐electrochemical microfluidic chip for EV detection. Reproduced with permission.^[^ ^290^ ^]^ Copyright 2023, Elsevier. e) An electrochemical strategy for EV detection assisted by Au nanoparticles (NPs). Reproduced with permission.^[^ ^291^ ^]^ Copyright 2023, Elsevier.

Following the exploration of computational methods for EV analysis, the discussion extends to other advanced technologies, like electrochemical sensing. Electrochemical sensors have garnered attention in EV research, promising sensitive and efficient EV detection crucial for point‐of‐care diagnostics and biosensing applications.^[^ ^285^ ^]^ Just as computational techniques have advanced SERS's analytical capabilities, electrochemical sensors have significantly benefited from the integration of advanced materials and novel design of devices.^[^ ^52^, ^286^ ^]^ One advancement comes from Li et al., who engineered an electrochemiluminescence (ECL) sensor for tumor‐related EV (**Figure** **9**a).^[^ ^287^ ^]^ This sensor uses a glycosyl‐imprinted polymer for dual recognition‐via glycan binding and aptamer interaction‐achieving remarkable sensitivity with a detection limit as low as 641 particles/mL across a broad concentration range. In a separate study, Hyungsoon and collaborators introduced a label‐free plasmonic sensing platform, enhancing detection sensitivity through electrophoretic and dielectrophoretic forces (Figure 9b).^[^ ^288^ ^]^ This innovative approach concentrates EVs on the sensor surface, allowing for the rapid detection of cancer‐related EVs in human plasma within 10 min, significantly increasing sensitivity. Another study by Revzin et al. presented an electrochemical immunoassay for urinary EV detection, utilizing a novel microwell plate. This method involves immobilizing EVs on electrode surfaces, tagged with gold nanoparticles that offer both target specificity through antibodies and electrochemical signaling through redox‐active metal ions. This enables precise analysis of specific markers, such as podocin and nephrin, in urinary cell vesicles (Figure 9c).^[^ ^289^ ^]^ Further contributing to the field, Yu et al. developed a microfluidic chip that integrates EV purification and protein detection, featuring a dual‐filter system and an electrode detection region enhanced with Zr‐MOFs (Figure 9d).^[^ ^290^ ^]^ This design streamlines the detection process, achieving a notably low detection limit. Concurrently, Wang et al. applied a gold nanoparticle‐assisted electrochemical strategy for the multiplex analysis of breast cancer‐derived EVs (Figure 9e).^[^ ^291^ ^]^ By using methylene blue‐functionalized HER2 aptamers and ferricyanide‐functionalized EpCAM aptamers anchored to gold nanoparticles, the technique distinguishes between HER2‐positive and HER2‐negative breast cancer EVs with high specificity and sensitivity. These evolving methodologies exemplify the substantial progress being made in the electrochemical sensing of EVs, further broadening the application of these technologies in clinical diagnostics and biosensing.

**Figure 10:**
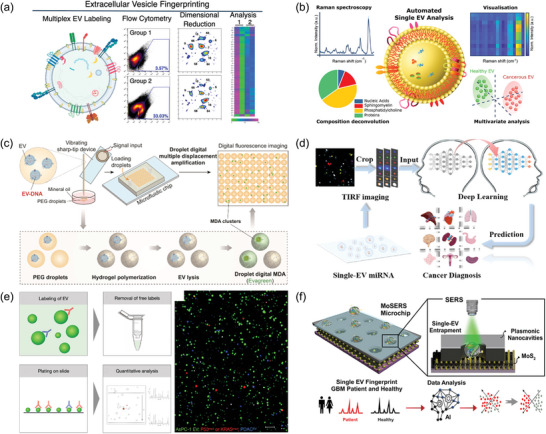
Innovative techniques for single‐EV research. a) Single‐vesicle flow cytometry with multiple parameters for the analysis of EV heterogeneity. Reproduced with permission.^[^ ^302^ ^]^ Copyright 2024, American Chemical Society. b) Single EV analysis using Single Particle Automated Raman Trapping Analysis platform (SPARTA). Reproduced with permission.^[^ ^303^ ^]^ Copyright 2021, American Chemical Society. c) Hydrogel droplet digital amplification for profiling DNA cargos in single EV. Reproduced with permission.^[^ ^304^ ^]^ Copyright 2024, American Chemical Society. d) A method to identify multi‐miRNA in single EV through the integration of Total Internal Reflection Fluorescence imaging and deep learning analysis. Reproduced with permission.^[^ ^305^ ^]^ Copyright 2024, American Chemical Society. e) Multiplexed protein measurements in single EV through fluorescence imaging. Reproduced with permission.^[^ ^306^ ^]^ Copyright 2022, American Association for the Advancement of Science. f) A microfluidic surface embedded with arrayed nanocavity microchips enables SERS detection of single EV from glioblastoma. Reproduced with permission.^[^ ^307^ ^]^ Copyright 2023, American Chemical Society.

Expanding on our discussion of EV analysis, it's vital to spotlight the emerging focus on single EV analysis. This cutting‐edge approach has rapidly gained prominence, attracting considerable interest across the scientific community.^[^ ^292^ ^]^ Traditional bulk analysis methods often fall short in dissecting the heterogeneity inherent in EV populations, thereby constraining our ability to fully unravel the complexities within EV populations.^[^ ^293^ ^]^ The emergence of single EV analysis techniques has overcome these challenges, offering researchers unparalleled precision to explore the distinct properties of individual EV. This breakthrough marks a new era in EV study, greatly enriching our understanding of EV heterogeneity and opening avenues for early disease detection and precision medicine.^[^ ^294^ ^]^ Recent technological advancements have introduced various methods for single EV analysis, such as nanoflow cytometry,^[^ ^250^, ^295^ ^]^ the ExoView platform,^[^ ^296^ ^]^ super‐resolution fluorescence imaging,^[^ ^297^ ^]^ SPR technology^[^ ^298^ ^]^ and single‐particle dark‐field imaging.^[^ ^299^ ^]^ We will examine the latest breakthroughs in this field, including plasma‐based techniques, microfluidics, single particle automated Raman trapping analysis (SPARTA), fluorescence imaging and the integration of artificial intelligence with SERS. Capturing nano‐sized EVs has posed significant challenges for traditional optical tweezers.^[^ ^300^ ^]^ Addressing this limitation, Ndukaife et al. introduced Geometry‐Induced Electrohydrodynamic Tweezers, enabling transport and capture of single EV near plasma hotspots without photothermal heating, thus maintaining low temperatures at capture points.^[^ ^301^ ^]^ This innovation opens new paths for downstream analysis of single EV. Very recently, Zijlstra et al. unveiled “EV Fingerprinting,” a technique for identifying unique vesicle populations through the dimensional reduction of multiparametric data from quantitative single‐EV flow cytometry (**Figure** **10**a).^[^ ^302^ ^]^ The method exploits shifts in fluorescence intensity and emission spectra of the lipophilic dye to analyze unique EV populations. Their research reveals that EV heterogeneity is not random but is governed by specific processes involved in EV biogenesis. Additionally, Stevens and their team created the SPARTA platform for high‐throughput exploration of EVs as biomarkers for breast cancer, as illustrated in Figure 10b.^[^ ^303^ ^]^ This system demonstrates the capacity for efficient and automatic isolation of individual EVs, achieving exceptional sensitivity and specificity (>95%). Furthering the scope of single EV analysis, Liu et al. unveiled a hydrogel droplet‐based digital multiplex displacement amplification technique for comprehensive EV‐DNA analysis, uncovering that 5%–40% of EVs carry DNA, with variations dependent on their cellular origin and size (Figure 10c).^[^ ^304^ ^]^ In an effort to enhance diagnostic precision, Chen et al. combined Total Internal Reflection Fluorescence imaging with deep learning for analyzing miRNA in single EV, achieving a 100% accuracy rate in predicting conditions for patients and healthy controls (Figure 10d).^[^ ^305^ ^]^ Similarly, Weissleder et al. developed a method for multiplex proteins measurement within single EV, facilitating the early detection of stage 1 pancreatic cancer (Figure 10e).^[^ ^306^ ^]^ Their research highlighted that ≈40% of EVs from original pancreatic cancer cells carrying KRAS and/or P53 mutations exhibited the same mutations. Concurrently, Mahshid et al. described a multiplexed microfluidic device with embedded nanocavity microchips for 97% encapsulation and SERS analysis of single EV, enhancing diagnostic accuracy to 87% and detecting glioma mutations in patient samples (Figure 10f).^[^ ^307^ ^]^ Another method for single EV analysis is electrochemical solution. Qian et al. explored an electrochemical approach with a nanopipette‐based method for the amperometric detection of individual exosomes and their dopamine content, providing a quantitative assessment of dopamine in individual EVs.^[^ ^280c^ ^]^ These advancements underscore the significant potential of single EV analysis techniques in advancing our understanding of EV biology and their application in disease diagnostics and precision medicine.

## 5 Challenges

Encased in a phospholipid bilayer, EVs constitute a group of vesicular entities. These vesicles are secreted by a wide variety of cell types and are consistently present in diverse biological fluids.^[^ ^10a^ ^]^ Possessing unique physicochemical and biochemical properties, EVs act as carriers for a multitude of bioactive compounds, such as proteins, nucleic acids, lipids and metabolites.^[^ ^30^, ^308^ ^]^ They play significant roles in various physiological and pathological activities, including cell‐to‐cell communication, immune system regulation, antigen presentation, cellular specialization, and the invasion of cancer cells.^[^ ^12^, ^309^ ^]^ Research also indicates that EVs have potential clinical application prospects in disease diagnosis, treatment, and as drug carriers. Despite the enormous research potential in the field, the number of FDA‐approved EVs‐based in vitro diagnostics and therapeutics remains limited. This situation highlights a lengthy path toward comprehensive clinical application and presents an array of challenges, broadly categorized into five major areas.

### 5.1 Preparation Obstacles in EV Research

During the EV preparation process, researchers encounter several critical challenges. Primarily, the high heterogeneity of EVs leads to significant differences in size, density, and composition, making the separation of specific subsets a complex task.^[^ ^58e^ ^]^ Moreover, the size and physicochemical properties of EVs overlap to varying degrees with non‐EV substances, such as lipoproteins and chylomicrons, further complicating the preparation process.^[^ ^11a^ ^]^ This variability not only complicates the precise separation of EVs but also introduces significant challenges in achieving a balance between efficiency and purity. Often, attempts to enhance one aspect result in compromises on the other, as seen with methods like polymer precipitation, where increased yield may come at the cost of reduced purity.^[^ ^310^ ^]^ Besides, the diversity of biological sample sources‐including blood, urine, and cell culture media‐requires customized processing approaches. Each type of sample demands its unique optimization strategy, further escalating the complexity of EV research.^[^ ^88^ ^]^ Additionally, mass production of EVs faces challenges such as ensuring consistent quality across batches, alongside the scalability of production methods.^[^ ^311^ ^]^ Addressing these challenges requires the development of more efficient separation technologies. A promising approach is the integration of various techniques‐such as ultracentrifugation, and size‐exclusion chromatography‐to bypass the limitations of individual methods and enhance both the purity and efficiency of EV preparation.^[^ ^116^ ^]^ Furthermore, employing reference materials for the quantifiable assessment of purification efficacy and specificity offers a strategic path to navigate the complexities of this field.^[^ ^312^ ^]^ Despite EVs' significant biomedicine potential, overcoming their preparation challenges demands ongoing research and technological innovation, emphasizing the need for constant methodological advancement.

### 5.2 Challenges in EV Analysis

The nanoscale size and complex, low‐abundance contents of EVs pose significant challenges for their biophysical and biochemical analysis. Biophysically, the challenge lies in the insufficient sensitivity of current detection techniques, which often leads to an underestimation of smaller EV subsets and results in an inaccurate assessment of their total number. The limited resolution of current techniques further complicates matters by inaccurately elevating particle concentration estimates, as it becomes difficult to differentiate EVs from other nanoscale materials.^[^ ^313^ ^]^ Biochemically, the detection and quantification of EV contents, such as proteins and nucleic acids, are hindered by their scant presence. For instance, on average, each extracellular vesicle may contain only one microRNA molecule, with one microRNA present per 100 EVs and one full‐length RNA molecule per 1000 EVs.^[^ ^314^ ^]^ This requires employing highly sensitive techniques for the detection and quantification of EV nucleic acids, essential for gaining valuable insights into their biological roles and associations with diseases. Labeling EVs for analysis, a crucial step for elucidating their biological roles, also faces hurdles. Despite various labeling techniques exist, including membrane fluorescent dyes (e.g., DiI, DiR, DiO, PKH26/67),^[^ ^315^ ^]^ bio‐conjugation (e.g., click chemistry),^[^ ^316^ ^]^ non‐covalent labeling (e.g., fluorescent antibodies or aptamers),^[^ ^317^ ^]^ bioluminescence (e.g., luciferase labelling),^[^ ^318^ ^]^ radiolabeling (e.g., iodine radioisotopes),^[^ ^319^ ^]^ and nanoparticle labeling (e.g., Quantum Dots),^[^ ^320^ ^]^ each approach comes with its own limitations. For example, lipophilic dyes can form aggregates due to their molecular properties, impacting experimental results.^[^ ^321^ ^]^ To address these analytical challenges, the development of sophisticated labeling techniques and the integration of cutting‐edge technologies with enhanced sensitivity and resolution are critical.^[^ ^322^ ^]^ An efficient approach is the in vivo fluorescence labeling strategy, which tags parent cells so that the EVs they release are also fluorescently marked.^[^ ^323^ ^]^ Additionally, advances in high‐resolution imaging, single‐particle analysis, and molecular analysis through high‐throughput sequencing and spectrometry are essential. These technologies will enable researchers to understand EVs' biological characteristics and potential clinical applications more accurately, thereby driving progress in the field of EV research.^[^ ^88^ ^]^

### 5.3 Absence of Standardization

In the rapidly evolving field of EV research, the lack of standardized methodologies poses significant challenges to both research integrity and clinical applications.^[^ ^324^ ^]^ EVs are increasingly recognized for their potential in diagnosing diseases, therapeutic interventions, and as drug delivery. However, the field's inherent complexity and the diverse nature of EVs transform standardization into a critical hurdle. Achieving uniformity is vital for ensuring the dependability of scientific findings and driving advancements in clinical practices.^[^ ^325^ ^]^ Standardization in EV research involves methodologies, quality control, and assessments of safety and efficiency. Various methods for EV preparation and analysis, each with its own benefits and drawbacks, underscores the need for precise controls and standard operating procedures to preserve EV integrity and functionality. Therefore, establishing methodologies is crucial to ensure consistency of results and comparability between different laboratories.^[^ ^92a^ ^]^ Quality control presents another intricate aspect of standardization, spanning all stages from cell selection to EV preservation.^[^ ^49a^ ^]^ For example, the condition of source cell impacts EV yield and properties, while slight changes in cultivation can affect EV phenotype and function.^[^ ^326^ ^]^ Incorrect processing or preservation might compromise EV integrity.^[^ ^327^ ^]^ Thus, establishing standardized guidelines, including protocols, quality metrics, and assessment methods, is crucial for the consistency and reliability of EV research and its applications. For EV‐based therapeutic strategies, rigorous evaluations of safety and efficacy are essential, requiring adherence to Good Manufacturing Practice (GMP) standards specifically tailored to the unique attributes of EVs. Assessing biodistribution, targeting efficiency, immunogenicity, and long‐term safety is crucial for developing comprehensive GMP guidelines and evaluation protocols that facilitate their clinical application.^[^ ^328^ ^]^ To tackle these challenges, organizations like the ISEV are working on standardized guidelines to enhance EV research transparency, reproducibility, and method optimization.^[^ ^329^ ^]^

### 5.4 Insufficient Comprehension of Underlying Mechanisms

Current research into biogenesis and cellular uptake of EVs remains inadequate. Although researchers have postulated a variety of biological mechanisms for the formation of EVs, consensus on the molecular sorting processes that govern EV cargos remains elusive.^[^ ^330^ ^]^ One of the major challenges is deciphering how EVs traverse the space between donor and recipient cells and the subsequent steps for cargos delivery and functional integration upon internalization.^[^ ^61^, ^330^ ^]^ Questions remain about the specifics of cargos loading within EVs, as well as the underlying principles that dictate the variability in EV size and composition. The regulatory factors that either promote or inhibit EV biosynthesis are not fully understood. This lack of understanding underscores the need for deeper insights into the fundamental biology of EVs, which is crucial for improving preparation and analysis techniques and potentially fast‐tracking the clinical applications of these vesicular structures.^[^ ^61^, ^331^ ^]^ To bridge these gaps, there is a call for interdisciplinary collaboration. By uniting experts from basic cell biology, clinical medicine, technology, and computer science, we can foster a more comprehensive understanding of EVs. This collaborative effort is vital for deciphering the complex mechanisms of EV biogenesis, cargos sorting, and uptake, pivotal for leveraging their therapeutic and diagnostic potential.

### 5.5 Advancing EV Research Rigor

To further enhance the rigor within the field of EV research, the International Society for Extracellular Vesicles (ISEV) has established a dedicated Rigor & Standardization Subcommittee (https://www.isev.org/rigor‐standardization).^[^ ^332^ ^]^ More recently, ISEV released the MISEV2023 guideline, addressing a wide range of pressing issues from nomenclature to in vivo studies, building on the efforts of MISEV2014 and MISEV2018.^[^ ^4^, ^333^ ^]^ This initiative has significantly promoted transparency and increased awareness among researchers regarding the rigor in EV studies. However, the degree of compliance with the MISEV guidelines and the utilization of additional voluntary online reporting platforms, such as EV‐TRACK, need further enhancement.^[^ ^325a^ ^]^ A survey revealed that 63% of researchers did not perform quality analysis before EV isolation, and 55% did not quantitatively analyze yield and contaminants.^[^ ^104^ ^]^ A 2021 survey conducted by the ISEV board found that about one‐third of respondents did not adhere to the guidelines or did not publish EV research after MISEV2018.^[^ ^334^ ^]^ Additionally, the survey indicated that 34% of the respondents were aware but had not used EV‐TRACK, while 28% were unfamiliar with this platform, which records experimental parameters for EV research, offering higher quality and transparency.^[^ ^197b^ ^]^ These surveys underscore the need for further raising awareness about rigor among researchers, especially those new to the field. Moreover, publishing complete datasets, detailed methodologies, and controls on platforms like EV‐Track can enhance the robustness and integrity of research in this evolving field.^[^ ^325b^ ^]^

## 6 Conclusion and Outlook

Over the past two decades, both the academic and industrial sectors have witnessed the burgeoning development of EVs. A plethora of high‐quality articles and patents are published and filed annually, coupled with an increasing number of approved sponsorships or investment projects dedicated to EVs.^[^ ^335^ ^]^ The enthusiasm surrounding EVs stems from their potential to revolutionize our understanding of disease biology, diagnosis, and therapeutic interventions. However, the complexity of EV biogenesis and their pronounced heterogeneity in size, composition, origin, and function pose significant challenges in their preparation and analysis.^[^ ^11^, ^68^ ^]^ Despite these challenges, UC continues to be the primary method for EV purification. Nevertheless, a growing contingent of researchers is exploring alternative methodologies, such as SEC and microfluidic platforms.^[^ ^104^ ^]^ As for analysis techniques, conventional methods such as NTA, TEM, and WB remain indispensable. Emerging technologies like novel SERS and electrochemical devices have significantly advanced the sensitivity and specificity of EV analysis. Additionally, instruments with higher resolution capabilities, such as single molecule localization microscopy, provide invaluable technical support for studies focused on single vesicles.^[^ ^297b^ ^]^ Microfluidics has been extensively reported as a pivotal tool for both EV preparation and analysis.^[^ ^51^, ^336^ ^]^ Yet, based on current research, microfluidic applications in EV processing still present four critical limitations: 1) the complexity of microfluidic device fabrication hampers scalability; 2) the propensity for channel clogging and non‐specific adsorption; 3) the need for improvement in sample throughput; and 4) a paucity of clinically validated examples, with most existing studies restricted to laboratory‐scale demonstrations.^[^ ^337^ ^]^ In light of these limitations, the integration of 3D printing technology in the development of microfluidic devices holds the potential to significantly advance EV research, offering innovative solutions to these existing limitations.^[^ ^338^ ^]^ After careful consideration, it is suggested that future developments in the field of EV preparation and analysis technologies should focus on four key areas:

### 6.1 High‐Throughput and Cost‐Effective Automated EV Processing Strategies

High‐throughput and cost‐effective automated strategies for EV preparation are emerging as a pivotal direction for future research in EV processing. These strategies aim to significantly enhance the efficiency and throughput of EV preparation through automated processes, reducing operational costs and minimizing human errors, thereby facilitating large‐scale production and application of EVs. This is particularly critical in the application of EVs as therapeutic agents and drug carriers.^[^ ^339^ ^]^ One illustrative example is the use of large bioreactors, such as hollow fiber bioreactors, for mass cell culture followed by EV collection, and utilizing techniques suitable for batch processing‐such as membrane‐based processes‐on automated platforms can achieve rapid, efficient separation of EVs with high purity and specificity.^[^ ^94^, ^311^ ^]^ Additionally, integrating cutting‐edge and highly sensitive online analytical technologies, such as real‐time flow cytometry and nanoparticle tracking analysis, enables the continuous monitoring of critical parameters. These parameters include EV size distribution, particle concentration, and surface markers. By incorporating these automated EV preparation strategies, future research will be able to uncover the potential of EVs more efficiently in disease diagnosis, treatment, and regenerative medicine, thus laying a solid foundation for their clinical application.

### 6.2 Single EV Multiparametric Analysis Techniques

The need for single EV multiparametric characterization techniques arises from the inherent heterogeneity of EVs.^[^ ^58e^ ^]^ Traditional methods of bulk analysis often miss this variability, limiting the depth of insights into individual EVs. This limitation impacts the accuracy of diagnostics and monitoring for various diseases, underscoring the importance of single EV analysis.^[^ ^294^ ^]^ This challenge has led to an increased focus on single EV analysis, inspiring the development of new methodologies. For instance, multiparametric single‐vesicle flow cytometry for analyzing EV heterogeneity and dynamic immunoassay for single tumor‐derived EV surface protein profiling via quantitative plasmonic imaging.^[^ ^302^, ^340^ ^]^ Despite these advancements, research into single EV remains in its nascent stages, with no existing method capable of simultaneously measuring the size of a single EV and providing a comprehensive analysis of its biological content. Another critical research direction is the in situ imaging and tracking of single EVs. This approach promises to provide deeper insights into cellular dynamics, including the biogenesis of EVs, intercellular transport, uptake, and cargos release.^[^ ^341^ ^]^ There is also a need to develop more efficient and robust multiplexed profiling for single EV, such as detecting target proteins and microRNAs with high sensitivity and specificity simultaneously, which could help advance the application of EVs in liquid biopsy.^[^ ^342^ ^]^ These studies not only deepen our understanding of EVs but also significantly enhance their potential applications in the medical field.

### 6.3 Multi‐Omics Integrative Analysis of EVs

Biomarkers encapsulated within EVs can serve as powerful indicators of pathological states in target cells or specific tissue types, thus enhancing the diagnostic precision for chronic or malignant diseases that conventional methods might miss.^[^ ^19^, ^343^ ^]^ Current research efforts focus on analyzing the contents of EVs to characterize their attributes or investigate their roles across a range of disease mechanisms through transcriptomic, proteomic, metabolomic, lipidomic, and genomic analyses.^[^ ^344^ ^]^ The utilization of omics data from EVs has been instrumental in identifying specific biomarkers for a variety of cancers, such as breast,^[^ ^206^ ^]^ lung,^[^ ^345^ ^]^ colorectal,^[^ ^346^ ^]^ and prostate cancers.^[^ ^347^ ^]^ However, studies confined to a single omics dimension offer limited insight into the intricate biological mechanisms of diseases, overlooking the complex interactions between DNA, RNA, proteins, and metabolites. These interactions, often complementary and synergistic, collaborate to perform specific biological functions. Therefore, adopting an integrative multi‐omics approach to study EVs is critically important for a comprehensive understanding of their functions, origins, and potential applications.^[^ ^344^ ^]^ While commercial entities (e.g., Mursla, https://mursla.com/science/) and extensive research efforts have started to delve into this area, there remains a significant need for deeper exploration.^[^ ^348^ ^]^ A pivotal future direction for EV research is the development of cutting‐edge computational methods (e.g., machine learning) and bioinformatics tools for multi‐omics analysis, which promise to reveal more profound insights into the compositional complexity and functional capacities of EVs.

### 6.4 Employment of Advanced Computer Algorithms for EV Data Analysis

The adoption of advanced computational algorithms for analyzing EV data represents a significant leap forward in the field.^[^ ^349^ ^]^ This approach leverages the formidable power of computational tools to interpret complex datasets, thereby enriching our understanding of the characteristics and functionalities of EVs. For instance, machine learning and artificial intelligence (AI) technologies can be employed to categorize EVs based on their biophysical and molecular attributes, predict their cargos contents, and even infer their potential biological effects.^[^ ^350^ ^]^ In a recent groundbreaking study, Zhao et al. introduced SEVtras, an algorithm for tracking heterogeneity in EVs.^[^ ^351^ ^]^ Additionally, computational algorithms facilitate the integration and analysis of multi‐omics data from EV research, including genomics, proteomics, transcriptomics, and metabolomics.^[^ ^271^, ^273^, ^274^ ^]^ This comprehensive analysis method can reveal the molecular composition of EVs, offering insights into their biogenesis, targeting mechanisms, and interactions with recipient cells.^[^ ^270^, ^352^ ^]^ Moreover, developing computational models to simulate EV dynamics‐including release, distribution, and uptake‐offers valuable insights into their roles in health and disease. Such models are instrumental in devising EV‐based therapeutic strategies, including the optimization of dosing schedules and administration routes.^[^ ^353^ ^]^ Advancing this research direction requires close collaboration between EV researchers and computational scientists. Establishing databases and repositories for storing and sharing EV data is essential for the development of algorithms and models, underscoring the significance of concerted efforts in this evolving field.

It should be acknowledged that the field of EVs remains nascent. Despite the considerable progress witnessed in recent years, numerous questions still remain unanswered. Among the most prominent challenges are the identification of specific biomarkers for various EV subgroups and the understanding of the mechanisms underlying the sorting of EV contents. To surmount these challenges, interdisciplinary collaborations hold substantial promise; by integrating translational medicine, clinical research, and industrial sectors, the path toward technological innovations and practical applications in the EV field becomes clearer. With ongoing efforts directed toward biomarker validation and regulatory compliance, the future appears promising for the approval of additional EV‐based diagnostic and therapeutic products. Such advancements hold the potential to revolutionize cancer prevention, diagnosis, and treatment through the deployment of precise and efficacious interventions. In summary, the prospects for EV applications shine brightly, yet navigating the journey ahead will require sustained and dedicated effort.

## Conflict of Interest

The authors declare no conflict of interest.
